# Celecoxib Nanoformulations with Enhanced Solubility, Dissolution Rate, and Oral Bioavailability: Experimental Approaches over In Vitro/In Vivo Evaluation

**DOI:** 10.3390/pharmaceutics15020363

**Published:** 2023-01-20

**Authors:** Aslıhan Arslan, Barbaros Yet, Emirhan Nemutlu, Yağmur Akdağ Çaylı, Hakan Eroğlu, Levent Öner

**Affiliations:** 1 Department of Pharmaceutical Technology, Faculty of Pharmacy, Hacettepe University, 06100 Ankara, Turkey; 2 Department of Cognitive Science, Graduate School of Informatics, Middle East Technical University, 06800 Ankara, Turkey; 3 Department of Analytical Chemistry, Faculty of Pharmacy, Hacettepe University, 06100 Ankara, Turkey

**Keywords:** celecoxib, dry co-milling, response surface methodology, central composite design, black-box, Bayesian optimization, intrinsic dissolution rate, characterization, pharmacokinetics

## Abstract

Celecoxib (CXB) is a Biopharmaceutical Classification System (BCS) Class II molecule with high permeability that is practically insoluble in water. Because of the poor water solubility, there is a wide range of absorption and limited bioavailability following oral administration. These unfavorable properties can be improved using dry co-milling technology, which is an industrial applicable technology. The purpose of this study was to develop and optimize CXB nanoformulations prepared by dry co-milling technology, with a quality by design approach to maintain enhanced solubility, dissolution rate, and oral bioavailability. The resulting co-milled CXB composition using povidone (PVP), mannitol (MAN) and sodium lauryl sulfate (SLS) showed the maximum solubility and dissolution rate in physiologically relevant media. Potential risk factors were determined with an Ishikawa diagram, important risk factors were selected with Plackett-Burman experimental design, and CXB compositions were optimized with Central Composite design (CCD) and Bayesian optimization (BO). Physical characterization, intrinsic dissolution rate, solubility, and stability experiments were used to evaluate the optimized co-milled CXB compositions. Dissolution and permeability studies were carried out for the resulting CXB nanoformulation. Oral pharmacokinetic studies of the CXB nanoformulation and reference product were performed in rats. The results of in vitro and in vivo studies show that the CXB nanoformulations have enhanced solubility (over 4.8-fold (8.6 ± 1.06 µg/mL vs. 1.8 ± 0.33 µg/mL) in water when compared with celecoxib pure powder), and dissolution rate (at least 85% of celecoxib is dissolved in 20 min), and improved oral pharmacokinetic profile (the relative bioavailability was 145.2%, compared to that of Celebrex^®^, and faster t_max_ 3.80 ± 2.28 h vs. 6.00 ± 3.67 h, indicating a more rapid absorption rate).

## 1. Introduction

Most of the newly discovered drugs and non-steroidal anti-inflammatory drugs (NSAIDs) which are frequently prescribed have low solubility, and therefore low oral bioavailability [1,2,3]. In recent studies, it has been aimed to improve the pharmacokinetic properties of drugs in order to ensure that NSAIDs, which are used continuously in the treatment of rheumatoid arthritis, osteoarthritis, and ankylosing spondylitis, are used at the lowest effective dose, and therefore to reduce their side effects [4]. To reduce the therapeutically effective dose of a drug, first, pharmacokinetic properties should be improved according to the reference dose. In order to improve the pharmacokinetic properties of these drugs, it is necessary to increase their bioavailability due to their solubility and dissolution properties [5]. Because, in cases where the dissolution rate of the drugs is slower than the absorption rate, the absorption and bioavailability of the drugs are functions of the dissolution rate. Especially in Class II (high permeability, low solubility) drugs, frequently prescribed NSAIDs, according to the BCS, the rate-limiting step in the absorption rate is the dissolution rate [5,6].

There are many different methods applied to increase dissolution, absorption, and thus in vivo efficacy, by the elimination of solubility problems of drugs with low bioavailability [7,8,9,10,11]. To increase the solubility and dissolution rate of the drugs, the surface area of the active substance in contact with the ambient liquid must be increased [12,13,14,15]. The methods applied for this purpose are forming cyclodextrin complexes, preparing solid dispersions, microemulsion preparation methods, and particle size reduction methods. Particle size reduction methods are divided into two groups, as mechanically applied methods, and particle size control methods by engineering. In the process of mechanical particle size reduction (micronization/nanonization), milling with jet mills, high energy ball mills, and planetary ball mills are applied, as well as high pressure homogenization methods. The methods in which the particle size is controlled by engineering are the cryogenic spraying method which is a creation of nano-structured amorphous particles with high porosity at very low temperatures, and crystal engineering, which is obtaining metastable polymorph, high energy amorphous forms, very fine particles by controlled crystallization process [12].

Among the methods mentioned above, the mechanical particle size reduction method is the most industrial applicable method. There are many drugs whose particle size is reduced, and their bioavailability is increased by this method [1,13,16,17,18]. The wet milling method, on the other hand, is used to obtain more effective and positive results to reduce the particle size. It involves a laborious and long process in the production of solid dosage forms because the particles produced by the wet method must be converted into solid state by a technique, such as spray drying or lyophilization [13,19].

The dry milling system can be used to facilitate the milling process, but in this case, the possibility of re-agglomeration of the milled particles arises. In recent studies, a dry milling method using excipients with stabilizer and surfactant functions is also preferred as an effective method [20].

With a technology called SoluMatrix Fine Particle Technology™, substances are milled by the dry milling method and submicron particles are obtained at the end of the milling process. It is a patented dry milling technology that reduces the particle size of poorly soluble pharmaceuticals to submicron levels, which are 10 to 200 times smaller than standard drug particles [20]. In addition, a special mixture of excipients assists in milling and protects the drug particles against further agglomeration. With this technology, the solubility and dissolution rate of three different NSAIDs, namely diclofenac, indomethacin, and meloxicam, which are Class II according to BCS and are very common and chronically used in the treatment of arthritis, were increased [21,22,23].

This study focuses on optimizing submicron/nano-sized celecoxib particles by using dry co-milling technology with a quality by design approach. The main contribution of this study is to identify the optimal factors for increased solubility and dissolution rates of celecoxib nanoformulations and to evaluate them in vitro and in vivo studies. This study also makes a methodological contribution by demonstrating that a state-of-the-art data-driven optimization technique called Bayesian Optimization (BO) can reduce the effort required for experiments compared to conventional experimental design methods.

The Central Composite Design (CCD) model is an integral part of Response Surface Methodology (RSM). It is a combination of statistical and mathematical models that reduce the number of experiments by choosing the best experimental conditions to reach the target product profile [24]. The CCD model is otherwise called a Box-Wilson Central Composite Design. In this design, the center points are eventually augmented with the group of “star points” that allows the estimation of curvature. The difference of this methodology from the factorial design is that the quadratic effects of the factors that allow the response curvature to be studied can also be determined [25]. In this way, the response variable can be better understood and optimized. These designs have very good symmetry and rotatability and allow maximum information to be obtained with minimum experimentation. Therefore, it is one of the RSM’s that is most widely used in the development of pharmaceutical products and the optimization of manufacturing processes. With CCD, the effect of 2 to 50 factors on response variables can be examined at five levels (−*α*, −1, 0, +1, +*α*) [26,27,28,29,30].

The CCD models and other conventional experimental design models plan the specific experiments that will be undertaken, their sequence, and the number of repetitions, before starting the experiments. Unlike CCD, BO uses an iterative and data-driven approach for selecting the experiments and optimizing the response variable [31]. BO iteratively determines which new experiments enables us to learn most about the subject based on the results of previous experiments. The number of experiments to conduct with BO is not fixed, the iterative optimization process stops when the optimal point is reached. BO is especially suited for optimizing black-box functions that are expensive to evaluate [31]. It has been shown to efficiently find the optimal point with small number of experiments in other domains including material science and machine learning [32].

Therefore, BO offer a promising approach for reducing the number of expensive experiments when formulating pharmaceutical products. Despite the potential benefits, BO has been rarely used in the pharmaceutical technology domain. Recently, Sano et al. [33] used BO on a simulated dataset about drug formulation experiments that was generated by an Artificial Neural Network (ANN) model trained from a previously completed formulation experiment. Sano et al. have not conducted any experiments based on BO; they used BO only on the simulated data. The use of BO with real time experiments for the optimization of celecoxib (CXB) nanoformulation is an innovative approach for the drug formulation domain.

In the present study, CXB was used as the active pharmaceutical ingredient. CXB is a selective cyclooxygenase-2 inhibitor, and one of the commonly used NSAIDs for the acute or chronic treatment of the signs and symptoms of rheumatoid arthritis, osteoarthritis, ankylosing spondylitis, and the management of pain [34,35,36]. CXB belongs to the BSC Class II because of its low aqueous solubility (5 µg/mL at 5–40 °C) and high permeability properties. Its solubility below pH 9.0 is independent of pH, and is higher at pH 12 [34,35,37]. Several pharmaceutical technologies have been used to increase the solubility and dissolution rate of CXB in the physiological pH media, such as solid dispersions [38,39,40,41,42], solid phospholipid dispersions [43], nanosuspensions [44,45], nano emulsions [46], lipid-silica hybrid [47], cyclodextrin inclusion complexes [48], nano micelles [49], and liposomal [50]. While these technologies can be effective, it may be difficult to stabilize and produce on a large scale. However, dry co-milling technology was used for three different NSAIDs on the market, and studies are on-going for prostate cancer treatment. The commercial products of CXB are Celebrex^®^ (Pfizer, New York, NY, USA), Concensi^®^ (Purple Biotech, Rehovot, Israel), Seglentis^®^ (Esteve, Barcelona, Spain) and Elyxyb™ (Biodelivery Sciences, Raleigh, NC, USA). Celebrex^®^ is a product in oral capsule form containing CXB in strengths of 50 mg, 100 mg, 200 mg, and 400 mg for use in the treatment of osteoarthritis, ankylosing spondylitis, juvenile rheumatoid arthritis, acute musculoskeletal pain, chronic pain, and post-operative pain. Concensi^®^ is a product in tablet form containing CXB and amlodipine besylate intended for the treatment of pain from osteoarthritis and hypertension with a combined product [51]. Elyxyb™ is a product in oral solution form in 25 mg/mL strength, indicated for the treatment of acute migraine with or without aura [36].

The present research work aimed to formulate CXB nanoformulations with a higher solubility and dissolution rate, which are the rate-limiting steps of oral absorption by dry co-milling process with the most favorable excipients. We mainly focused on the design of experiment approach to understand the influence of process conditions and formulation parameters on the production of co-milled CXB compositions and its critical quality attributes (CQAs). Plackett-Burman design was used for the screening studies, and CCD was used for optimizing co-milled CXB composition. BO was used to optimize the co-milled CXB composition with reduced numbers of experiments compared to the known response surface methodology (CCD). Finally, the optimized co-milled CXB composition was characterized physiochemically, formulated as a capsule dosage form, the dissolution rate of optimum formulation versus reference product (Celebrex^®^) was tested, and pharmacokinetics properties were investigated in rats. The next goal of our study is to develop a low-dose CXB formulation compared to Celebrex^®^ to provide effective treatment in rheumatoid arthritis, osteoarthritis, ankylosing spondylitis and pain management. Therefore, we compared our in vitro and in vivo results with the Celebrex^®^.

## 2. Materials and Methods

### 2.1. Materials

CXB 4-(5-(4-methylphenyl)-3-(trifluoromethyl)-1*H*-pyrazol-1-yl) benzene sulfonamide (lot CE0311116) was purchased from Hetero Drugs Limited, Hyderabad, India. Povidone (PVP, Plasdone™ C-12, K-29/32, K-90), vinylpyrrolidone and vinyl acetate copolymer (PVP-VA, Plasdone™ S-630), and hydroxyethyl cellulose (Natrosol™ 250) were kindly donated by Ashland, İstanbul, Turkey. Sodium lauryl sulfate (SLS, Kolliphor^®^ SLS) and polyvinyl caprolactam-polyvinyl acetate-polyethylene glycol graft co-polymer (Soluplus^®^) was donated by BASF, İstanbul, Turkey. Mannitol (Pearlitol^®^ 100 SD), low-substituted hydroxypropyl cellulose (L-HPC LH-21), magnesium alumino metasilicate (Neusilin^®^), polyoxyl 40 stearate (Myrj™ S40) and lactose monohydrate (FlowLac^®^ 90) were donated by Barentz Chemical (İstanbul, Turkey), Harke Pharma (İstanbul, Turkey), Fuji Chemical (Tokyo, Japan), Croda (Snaith, UK) and Meggle (Wasserburg am Inn, Germany), respectively. Croscarmellose sodium (Ac-Di-Sol^®^) and magnesium stearate (Ligamed^®^ MF-2-V) were donated by IMCD, İstanbul, Turkey. Hard gelatin capsules were purchased from Lonza, New Jersey, USA. Celebrex^®^ 200 mg Capsules (Pfizer Inc., New York, NY, USA, lot C080351) were purchased commercially from Turkey. Biorelevant dissolution media powder (FaSSIF/FeSSIF/FaSSGF) was purchased from Biorelevant Ltd. (London, UK). Caco-2 cells (human colon carcinoma cell line) were purchased from American Type Culture Collection (Manassas, VI, USA). Thiazolyl blue tetrazolium bromide (MTT) was purchased from AppliChem GmBH (Darmstadt, Germany). Dulbecco’s Modified Eagle’s Medium (DMEM) and fetal bovine serum (FBS) were provided from BiochromAG (Berlin, Germany), and Penicillin-Streptomycin solution from Life Technologies, Inc., (Carlsbad, CA, USA). Thincerts™ cell culture inserts (transparent membrane, pore size: 0.4 µm, growth area: 1.13 cm^2^) were obtained from Grenier Bio-one (Frickenhausen, Germany). The water used in all experiments was ultra-purified water (Mili-Q, Millipore, Turkey). All analytical chemicals, buffer salts and reagents were all analytical grade and purchased from Merck KGaA (Darmstadt, Germany).

Male Sprague-Dawley rats (8–10 weeks old, 280 ± 20 g) were purchased from Kobay Laboratory Animals Center (Ankara, Turkey). All experiments were performed according to the guidelines of the Declaration of Helsinki and Turkish Law for the Protection of Animals and animal experimentation was approved by the local ethics committee of Kobay Laboratory Animals Center (approval number: 439, date: 2 December 2020).

### 2.2. Preparation of Co-Milled Celecoxib Composition and Physical Mixture (PM)

The excipients and CXB were individually weighed and mixed at different ratios, determined with the experimental design study described in Section 2.4. They were sifted from 1 mm sieve and were mixed in a small cylinder container for 10 min. The powder mixture was loaded into the agate milling jar (250 mL) with agate balls (16 mm) and milled by using a laboratory scale milling (Planetary Ball Mills PM 100, Retsch, Haan, Germany). The milling prosses was performed according to the milling speed, milling time and ratio of ball weight to powder weight as explained in Section 2.4. The temperature of milling was not controlled. The physical mixture (PM) containing CXB, and excipients was prepared in the same ratio as optimized co-milled CXB composition. All the powders were sifted from 1 mm sieve and were mixed in a small cylinder container for 10 min. The obtained co-milled CXB composition and PM were stored in amber glass bottles for further analysis. Each trial was performed in triplicate.

### 2.3. Preliminary Screening Experiments

The most suitable excipients for dry milling with CXB were determined as a result of preliminary screening experiments. CXB was milled alone and together with auxiliary substances with different chemical properties. The analysis of average particle size, intrinsic dissolution rate in pH 12 and pH 1.2 + 0.2% SLS and polymorphic structure were evaluated for selection of excipients (Table S1: Dissolved amount of CXB in pH 12 and average particle size results; Table S2: Dissolved amount of CXB in pH 1.2 + 0.2% SLS; Figure S1: XRD diffractograms for CXB, F01, F03, F20, and F21; Figure S2: FTIR spectra for CXB and F03). Based on the results obtained, it was decided to investigate the milling compositions containing CXB, Povidone (PVP), mannitol (MAN) and sodium lauryl sulfate (SLS) in detail with an advanced experimental design. Dry co-milling was carried out in a planetary ball mill. Considering the working principle of planetary ball milling, milling speed, milling time, ball size, filling ratio of powder and ratio of ball weight to powder weight are important parameters for milling success [52]. An Ishikawa diagram was established for the risk identification of the co-milling composition and milling process parameters, and to understand their potential effects on the physicochemical properties of co-milled CXB (Figure S3: Ishikawa diagram of CXB co-milling process). A six factor, two-level Plackett-Burman experimental design was used to statistically investigate the effects of selected formulation and process variables (Table S3: Plackett-Burman design and results, Table S4: Statistical analysis of dependent variable values obtained by Plackett-Burman experimental design).

### 2.4. Experimental Design and Optimization

#### 2.4.1. Central Composite Design (CCD)

The three major significant independent variables selected from Plackett–Burman affecting the response were PVP/CXB weight ratio (X_1_), SLS/CXB weight ratio (X_2_) and MAN/CXB weight ratio (X_3_). The low and high levels of factors were taken directly from the Plackett-Burman design, and the medium levels were established at the midpoint between the low and high levels. The factors and their levels used in CCD were summarized in Table 1. Twenty experimental runs were performed and thoroughly assessed by dissolved amount in pH 12 at 30 min (Y_1_), dissolved amount in pH 1.2 + 0.2% SLS at 120 min (Y_2_) as CQAs (response variables). Design Expert^®^ 13 (Stat-Ease Inc., Minneapolis, MN, USA) software was used for data treatment and response surface plots generation. Twenty experimental runs were obtained from the CCD matrix generated from Design Expert^®^ 13 consisting of 8 cube points, 4 center points in cube, 6 axial points, and 2 center points in axial with 2 blocks. The milling speed (250 rpm), milling time (1 h) and ratio of ball weight to powder weight (15:1) were kept constant as their effects on the response variables seemed statistically insignificant according to the results of Plackett–Burman design.

For model validation and to determine the optimum co-milled CXB, three different experiments offered by response surface model were made. Formulations were created and tested to figure out the correlation between expected and actual response levels. The optimum composition was further characterized for its CQAs.

#### 2.4.2. Bayesian Optimization (BO)

BO has two main elements: a *surrogate model* of the objective function that will be optimized and an *acquisition function* that identifies the next experiments. Gaussian Process (GP) models are often used as surrogate models for BO as they can be used to tractably calculate the posterior distributions over objective functions [53,54]. A GP can be interpreted as a generalization of the Gaussian distribution that defines a distribution over functions. In order to start BO, the surrogate model needs to be initialized and updated with the results of a set of initial experiments. After initializing the surrogate model, an acquisition function is employed to propose and evaluate new experiments that are likely to update the surrogate model effectively. Acquisition functions aim to balance the trade-off between exploring fewer known data points that may likely to lead to worse results and exploiting better known data points with high expected results. Expected Improvement (EI) and Confidence Bound (CB) are widely used acquisition functions for BO [53]. At each iteration, inputs with maximum acquisition function values are selected, experiments for those inputs are conducted, and the surrogate model is updated with their results. These iterations continue until a stopping rule is satisfied.

Multi-objective BO can be used if multiple response variable of interest needs to be optimized simultaneously [55]. In this study, we focused on optimizing a single variable of interest (Y_2_) with BO and compared its results with CCD to demonstrate the potential use of BO for drug formulation. Among the formulation variables used in BO, scans were made in the range of 0.5–2.0 for X_1_ and X_3_, and in the range of 0–0.2 for X_2_. The output value to be optimized was determined as dissolved amount in pH 1.2 + 0.2% SLS at 120 min. The aim of the BO experiment design was to maximize the amount of dissolved CXB.

MLRMBO package in R statistical software was used for BO [52]. A GP with the covariance function BO Matern 3/2 was used as the surrogate model for BO. BO was used to optimize the amount of dissolved CXB (Y_2_). This surrogate model was initialized using the 8 experiments given in Table 2. These initial experiments were randomly selected from CCD to avoid any biased selection that may be advantageous or disadvantageous for the BO results.

After initializing the surrogate models with the data shown in Table 2, the next 2 experiments to conduct were determined based on the EI and CB acquisition functions. These are coded as BO01 and BO02 in Table 3. Intrinsic dissolution rate tests of BO01 and BO02 coded formulations were performed, and Y_2_ response variable value was determined. The surrogate model was updated with the results of these BO01 and BO02, and the next two experiments BO03 and BO04 were suggested by the acquisition functions. These steps of obtaining next experiments from the acquisition function and updating the surrogate results of those experiments continued iteratively for a total of 6 iterations and 12 experiments (see Table 3 for a list of those experiments and Figure S4 for a flowchart of this process).

### 2.5. Characterization of Co-Milled Celecoxib Composition and Physical Mixture

#### 2.5.1. Determination of Solubility

The apparent solubility values of optimized co-milled CXB composition and PM were employed by the shake-flask method [56]. An excess amount of drug (equivalent to 10 mg CXB) was added to Eppendorf tubes containing 10 mL of water, pH 1.2 + 0.2% SLS, pH 4.5 (0.05 M acetate buffer contains sodium acetate and acetic acid) + 0.2% SLS and pH 6.8 (phosphate buffer contains dibasic sodium phosphate heptahydrate and monobasic sodium phosphate monohydrate) + 0.2% SLS as triplicate replicates. The tubes were vortexed and placed in a water bath at 37 ± 0.5 °C and shaken vigorously for 24 h. Samples were withdrawn after 24 h and filtered through 0.45 μm PTFE (Millex LG, Milipore, Billerica, MA, USA) filter. The filtrate was analysed by using HPLC for quantification procedure.

#### 2.5.2. Determination of Intrinsic Dissolution Rate (IDR)

IDR studies were carried out with Sotax AT 7smart Dissolution Testing System (Sotax CH-4123, Aesch, Switzerland) by using USP Wood Apparatus. The samples were accurately weighted and compressed by a hydraulic press of 600 psi pressure with dwell time of 30 s, using a punch having diameter of 0.8 mm, and surface area of 0.5 cm^2^ (Figure S5: USP Wood Apparatus components, sample preparation). The IDR experiments were carried out in 500 mL pH 12 (0.04 M tribasic sodium phosphate buffer contains trisodium phosphate dodecahydrate) at 100 rpm speed, and in 500 mL pH 6.8, pH 4.5, pH 1.2 with 0.2% SLS and FASSIF medium at 200 rpm speed at 37 ± 1 °C. The dissolution medium of 5 mL was withdrawn at predetermined time points of 1, 2, 3, 4, 5, 15, 30, 45, 60 for pH 12, and 3, 6, 9, 12, 15, 30, 60, and 120 min for other media from the vessels and the initial volume of the medium was maintained constant by adding 5 mL of the fresh dissolution medium to each of the vessels. Each sample was filtered through a 0.45 μm PTFE filter into the test tubes to remove undissolved CXB. After discarding the first 2 mL the subsequent filtrate was analysed by HPLC to quantify CXB over three replicates.

#### 2.5.3. Differential Scanning Calorimetry (DSC)

DSC thermograms were analysed by using differential scanning calorimeter (DSC 3, Mettler Toledo, Greifensee, Switzerland). Each sample was accurately weighed within 5–8 mg individually and placed in an aluminum pan, which was then covered with an aluminum lid. Before measurement, a heating process was done over the temperature range from ambient temperature (20 °C) to 200 °C at a scanning rate of 10 °C/min under nitrogen purge at 30 mL/min. DSC was pre-calibrated for baseline using an empty aluminum pan. All measurements were carried out in duplicate, and results analyzed using Star^e^ Excellence software.

#### 2.5.4. Fourier Transform Infrared Spectroscopy (FTIR)

FTIR analysis was performed with Agilent Cary 630 FTIR spectrometer (Agilent, Santa Clara, CA, USA) in the wavenumber range of 650–4000 cm^−1^, at 4 cm^−1^ resolution, and 3 scans were performed for each sample. Samples were analyzed directly without any sample preparation.

#### 2.5.5. Powder X-ray Diffraction Analysis (PXRD)

XRD patterns were collected using Rigaku Ultima-IV Ray Diffractometer (Rigaku, Austin, TX, USA) with Cu radiation operated at 40 kV and 30 mA. Data were obtained in the range of 3–35° at 1°/min scan speed and 0.02° sample width. Results were analyzed using XG Operation PINT 2200 software.

#### 2.5.6. Determination of Particle Size

The mean particle size (Z-average) distributions of co-milled CXB composition and PM were measured by dynamic light scattering (DLS) technique using a Zetasizer Nano ZSP system (Malvern Instruments, Malvern, UK) at room temperature. The required powder amount to equivalent 30 mg CXB was weighed and added to 10 mL purified water containing 0.1% (*w*/*v*) polysorbate 80. This suspension was homogenized by ultrasonic homogenizers (Sonopuls, Bandelin, Berlin Germany) for 3 min. Folded capillary zeta cell (DTS1070) was used for particle size measurement. Each sample was measured in triplicate.

#### 2.5.7. Scanning Electron Microscopy (SEM)

The average particle size, size distribution and morphology of CXB, co-milled CXB compositions, and PM surface characteristics were observed using a scanning electron microscope (Tescan GAIA FIB-SEM, Brno-Kohoutovice, Czech Republic). The samples were mounted on an aluminum stage using adhesive carbon tape and coated with gold under an argon atmosphere in a high vacuum evaporator.

#### 2.5.8. Storage Stability Study

The accelerated stability (40 °C ± 2 °C/75% ± 5% RH) and long-term storage condition (25 °C ± 2 °C/60% ± 5% RH) stability studies were carried out to investigate the physicochemical stability of optimized co-milled CXB composition for 3 months. The powder samples analyzed at 1st, 2nd and 3rd months for accelerated stability and 3rd month for long-term storage condition. Particle size, PDI, zeta potential, XRD, IDR and assay of optimized co-milled CXB composition and PM were determined evaluate the physicochemical stability.

### 2.6. Analytical Methods (High-Performance Liquid Chromatography (HPLC))

CXB quantification was carried out using a high-performance liquid chromatography (HPLC) instrument (Agilent, 1200 series LC, Santa Clara, CA, USA) system with a C18 column (250 mm × 4.6 mm, 5 μm particle size). Methanol and water (75:25 *v*/*v*) mixture was used as the mobile phase at a flow rate of 1 mL/min at 30 ± 0.5 °C. The UV detector was set at 254 nm and the injection volume was set as 20 μL. The chromatograms were evaluated with Chem Station Software (Agilent, ABD). CXB concentration was calculated using calibration curve consisted with seven different standards of CXB (R^2^ = 0.999). All samples were measured in triplicate. 

### 2.7. Formulation Studies

The CXB nanoformulations containing co-milled CXB was prepared by direct blending method. Co-milled CXB, croscarmellose sodium and magnesium stearate were mixed in the blender (Glatt CML 10 Container Blender, Glatt, Binzen, Germany) and then filled into the hard gelatin capsules manually (Table S5: Celecoxib Nanoformulation and Celebrex^®^ capsule qualitative and quantitative composition).

### 2.8. In Vitro Dissolution Study

The in vitro dissolution study was carried out using the dissolution equipment Sotax AT 7smart Dissolution Testing System (Sotax CH-4123, Aesch, Switzerland) and USP Apparatus II (Paddle). The dissolution conditions consisted of a medium volume of 1000 mL per vessel with a paddle rotation speed of 50 rpm. The temperature in the vessels was 37 ± 0.5 °C throughout each dissolution run. Different dissolution media of pH 12, pH 6.8, pH 4.5, pH 1.2 with different SLS concentrations such as 0.2% and 0.5% were used throughout the experiments. The dissolution medium of 5 mL was withdrawn at predetermined time points of 10, 20, 30, 45 and 60 min from the vessels and the initial volume of the medium was maintained as constant by adding 5 mL of the fresh dissolution medium to each of the vessels. The CXB nanoformulations was filled in the capsules to keep CXB amount of 37 mg for maintained sink conditions and 200 mg for non-sink conditions. Also, Celebrex^®^ capsule was used directly for non-sink conditions and Celebrex^®^ capsule composition was filled in the capsules to keep celecoxib amount of 19 mg for maintained sink conditions. A wire helix sinker was used to prevent the capsule from floating. The volume withdrawn was approximately 5 mL for each sampling time point. Each sample was filtered through a 0.45 μm PTFE filter (Millex LG, Milipore, Billerica, MA, USA) into the test tubes to remove undissolved CXB. After discarding the first 2 mL, and the subsequent filtrate was used to determine the CXB concentrations to determine with HPLC. Experiments were conducted at least in triplicate.

### 2.9. Cell Culture Studies

The cell culture studies were conducted for investigating cytotoxicity and permeability of pure CXB, CXB nanoformulations, Celebrex^®^ capsule, and PM. For this purpose, human colon carcinoma cell line (Caco-2 cells) was used. DMEM supplemented with FBS (10%, *v*/*v*), 2 mM L-glutamine, penicillin (50 U/mL) and streptomycin (50 µg/mL) was used as growth medium. Caco-2 cells were maintained in a humidified 5% CO_2_ air atmosphere at 37 °C.

#### 2.9.1. Cell Viability Studies

Thiazolyl blue tetrazolium bromide (MTT) analyses were carried out on the Caco-2 cell line (passage number: 22–26) to determine the cytotoxicity of pure CXB, CXB nanoformulation, Celebrex^®^ capsule, and PM. Cells were suspended in the growth medium and seeded in 96-well plates at 5 × 10^3^ cells/well. After 24 h, drug suspensions of pure CXB, optimized co-milled CXB, Celebrex^®^ capsule, and PM were added to the wells at different concentrations (1, 5, 10, 50, 100, 200, 500 and 1000 µM) and incubated for 4 h in a humidified 5% CO^2^ air atmosphere at 37 °C (n = 6). DMSO was used to solubilize the drug; therefore, DMSO solution (0.25–4%) in the growth medium was also applied as a control. After the incubation period, 25 μL MTT (5 mg/mL) solution was added to wells and incubated for more 4 h at 37 °C. Then the medium was removed and 200 μL of DMSO was added to each well to solubilize the formazan crystals. Absorbance was measured with a microplate reader (VERSAmax Molecular Devices Corporation, Sunnyvale, CA, USA) at 570 nm to assess cell viability. The absorbance was assumed to be 100% for the control group. Cell viability (%) was calculated according to Equation (1)).

(1) $$\mathrm{Cell}\;\mathrm{viability}\;\left(\%\right)=\frac{\mathrm{mean}\;\mathrm{absorbance}\;\mathrm{of}\;\mathrm{each}\;\mathrm{group}-\mathrm{mean}\;\mathrm{absorbance}\;\mathrm{of}\;\mathrm{blank}}{\mathrm{mean}\;\mathrm{absorbance}\;\mathrm{of}\;\mathrm{negative}\;\mathrm{control}-\mathrm{mean}\;\mathrm{absorbance}\;\mathrm{of}\;\mathrm{blank}}\times 100$$

#### 2.9.2. Permeability Studies

The permeability studies were conducted using Caco-2 cells for investigating permeability of CXB from pure CXB, CXB nanoformulations, Celebrex^®^ capsule, and PM. Harvested Caco-2 cells were counted with trypan blue and seeded at 100,000 cells/well on 12-well inserts having a pore size of 0.4 µm, and incubated in an incubator (Sanyo, Osaka, Japan) under 5% CO_2_ atmosphere at 37 °C. For 21 days, the medium was changed every other day. The integrity of the Caco-2 cell monolayer was assessed at the conclusion of the incubation period by measuring Transepithelial Electrical Resistance (TEER) with a Millicell-ERS voltameter (Millipore Sigma, Burlington, VT, USA). The inserts having 400–600 Ωcm^2^ resistance were used for transport studies.

Culture medium was replaced from each well by 500 µL and 1500 µL transport buffer (TB) (HBSS containing 10 mM HEPES (pH 7.4)) in the apical and basolateral sides, and the cell monolayers were subsequently equilibrated for 30 min at 37 °C. After removing the TB, suspensions of pure CXB powder, CXB nanoformulations, Celebrex^®^ capsule, and PM in TB were added to the apical side, while the basolateral side was filled with TB (n = 3). Plates were placed in a horizontal shaker and incubated at 37 °C for 2 h at 60 rpm. Samples were isolated from the basolateral sides (1.5 mL) 2 h after the incubation and analyzed with the HPLC.

The apparent permeability coefficients (P_app_, cm/sn) were calculated according to (Equation (2)).

(2) $$P_{\mathrm{app}}=\frac{\frac{\mathrm{dQ}}{\mathrm{dt}}}{A\times C_{0}}$$

where P_app_ is an apparent permeability coefficient, dQ/dt (μmol/L.min) is the cumulative amount of CXB which has been transported over the membrane, A (1.13 cm^2^) is the surface area of the inserts and *C*_0_ (μmol/L) is the initial concentration of the CXB on the apical site.

### 2.10. In Vivo Oral Bioavailability

The pharmacokinetic study was carried out to compare the oral bioavailability of Celebrex^®^ capsule to CXB nanoformulations, following oral administration in rats (10 and 40 mg/kg). The animals were housed in laminar flow, three per cage, and were kept at 22 ± 2 °C and 50–60% relative humidity for 1 week. The rats were fasted overnight (14–18 h) before drug administration. Twenty eight rats were divided into four groups: the rats in the first group received a dose of 10 mg/kg Celebrex^®^ capsule (n = 8), the second group 10 mg/kg CXB nanoformulation (n = 8), the third group 40 mg/kg Celebrex^®^ capsule (n = 6), and the fourth group 40 mg/kg CXB nanoformulation (n = 6), For oral administration, all formulations of Celebrex^®^ capsule powders and CXB nanoformulation powder were suspended in purified water by vortexing for 5 min. After drug administration, approximately 1.5 mL of blood samples were collected in heparinized tube from the teil vein at 0.5, 1, 2, 3, 4, 6, 12, and 24 h (Figure S6: Images of drug administration (A) and tail vein blood collection (B and C) in rats.) The blood samples were centrifuged at 10,000 rpm for 10 min at ambition condition. The plasma samples (about 250 μL) were stored at −70 °C. 200 μL acetonitrile including atorvastatin (internal standard) was added in 100 μL plasma, to precipitate proteins. The samples were vortexed at 30 s and centrifugated at 15,000 rpm for 15 min. The supernatant was separated and analyzed with liquid chromatography–tandem mass spectrometry (LC-MS/MS, Shimadzu 8030) to determine the amount of CXB in plasma samples. The LC-MS/MS method was adopted from Kim H-I et al. with slight modifications [57]. The method details were given in the supplementary section (Table S6: Chromatographic conditions for the celecoxib assay method; Table S7: MS/MS detection conditions for the celecoxib assay method). Eight different concentrations of calibration solutions, in the range of 0.005–10 μg/mL, were prepared with CXB and the internal standard, atorvastatin. The pharmacokinetic parameters including area under the curve (AUC_0–24h_, AUC_0–∞_, elimination half-life (t_1/2_), mean residence time (MRT), the elimination rate constant (k_e_), absorption rate constant (k_a_) were calculated by Phoenix WinNonlin (Version 8.1, Certara L.P., New Jersey, USA). Maximum concentration (C_max_) and time to reach maximum concentration (t_max_) were obtained from plasma concentration-time curve.

## 3. Results and Discussion

### 3.1. Optimization of Co-Milled CXB Composition Using CCD

After evaluation analysis of variance (ANOVA) results of Plackett-Burman design, the critical formulation and process variables were determined. As a result, it was concluded that the ratios of PVP/CXB, SLS/CXB, and MAN/CXB, are the critical formulation parameters that affect the critical quality properties of CXB. The low levels of milling speed and milling time provided optimum process conditions to increase amount of dissolved CXB. Because of that, milling time and milling speed were kept as 1 h and 250 rpm, respectively. Additionally, the ratio of ball weight to powder was kept at 15:1, because at high levels of ratio of ball weight to powder, the particle size resulted in the lowest value and dissolved amount resulted in as the highest value. To understand the effects of these three independent variables on the response variables, a CCD was applied, which included three factors, five levels, two blocks, eight cube points with an alpha value of 1.633, four center points on the cube, six axis points, and two center points on the axial. Twenty experimental runs were conducted in this study and six of them were zero point to validation model. The results of experimental runs are summarized in Table 4.

The quadratic model was found to be the best fitting model for the relation between independent factors and response variables after model selection for response analysis. The fit summary for each response was listed in Table 5. Following a multiple linear regression analysis of the data, the following polynomial equations were constructed to describe the quantitative impact of the analyzed independent variables and their interactions on the responses (Equations (3) and (4)):

(3) $$\mathrm{Dissolved}\;\mathrm{CXB}\;\mathrm{amount}\;\mathrm{in}\;\mathrm{pH}\;12\;(Y_{1})\;=\;-7.3\;-\;7.0\;\times \;X_{1}\;-\;157.8\;\times \;X_{2}\;\;+\;26.1\;\times \;X_{3}\;+\;250.5\;\times \;X_{1\times 2}\;+\;2.7\;\times \;X_{1}X_{3}\;-\;105.0\;\times \;X_{2}X_{3}\;+\;12.8\;\times \;\;{X_{1}}^{2}\;+\;608.9\;\times \;{X_{2}}^{2}\;-\;1.7\;\times \;{X_{3}}^{2}$$

(4) $$\mathrm{Dissolved}\;\mathrm{CXB}\;\mathrm{amount}\;\mathrm{in}\;\mathrm{pH}\;1.2\;+\;0.2\%\;\mathrm{SLS}\;(Y_{2})\;=\;-14.8\;+\;16.1\;\times \;X_{1}\;+\;\;1.8\;\times \;X_{2}\;+\;37.0\;\times \;X_{3}\;+\;188.2\;\times \;X_{1}X_{2}\;+\;0.25\;\times \;X_{1}X_{3}\;-\;4.3\;\times \;X_{2}X_{3}\;-\;\;7.79\;\times \;{X_{1}}^{2}\;-\;309.2\;\times \;{X_{2}}^{2}\;-\;19.19\;\times \;{X_{3}}^{2}$$

The most significant factor on the dissolved amount in pH 12 at 30 min (Y_1_) was ratio of the PVP/CXB. In this design, the *p* value of X_1_, X_2_, X_3_, X_1_X_2_, X_2_ X_3_ and X_2_^2^ were <0.05, and these were significant model terms as given in Table 5. Additionally, lack of fit value was not significant since *p* value and R^2^ were calculated as 0.1829 and 0.9706, respectively. Non-significant lack of fit and R^2^ values close to 1 meaning that the model fit was assessed using the coefficient of determination between the observed and the predicted values, consequently this model can be used navigate the design space [58,59].

The most significant factor on the dissolved amount in pH 1.2 + 0.2% SLS at 120 min (Y_2_) was ratio of SLS/CXB. The ratio of MAN/CXB was not significant statistically, however, the quadratic effect had significant influential effect on dissolved amount in pH 1.2. Also, lack of fit value was not significant due to *p* value of it was 0.2212 and the R^2^ value was 0.9216. Non-significant lack of fit and R^2^ values close to 1 meaning that the model fit was assessed using the coefficient of determination between the observed and the predicted values, consequently this model can be used navigate the design space [58,59].

**Figure 1:**
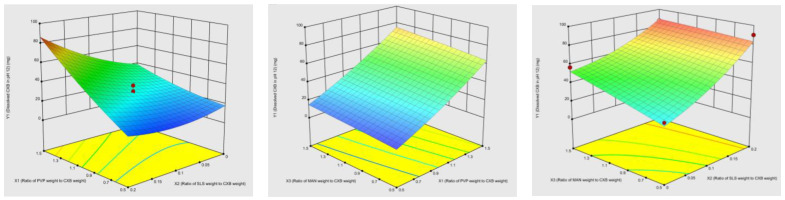
Three-dimension response surface showed the effects of PVP/CXB weight ratio (X_1_), SLS/CXB weight ratio (X_2_) and MAN/CXB weight ratio (X_3_) on dissolved amount in pH 12 after 30 min (Y_1_).

**Figure 2:**
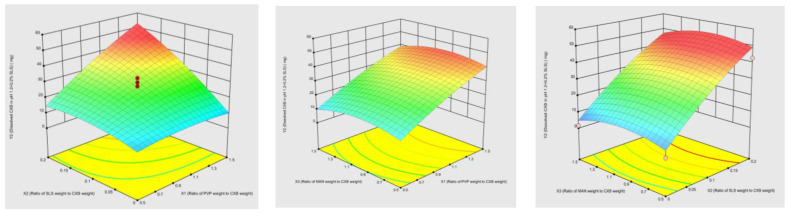
Three-dimension response surface showed the effects of PVP/CXB weight ratio (X_1_), SLS/CXB weight ratio (X_2_) and MAN/CXB weight ratio (X_3_) on the dissolved amount in pH 1.2 + 0.2% SLS after 120 min (Y_2_).

The three-dimension response surface and contour plots (Figure S7: Contour plots for Y_1_, Figure S8: Contour plots for Y_2_) were used for evaluation of the significant factors effect on the response variables. The effect of the interactions between different formulation factors on dissolved amount in pH 12 at 30 min (Y_1_) and the dissolved amount in pH 1.2 + 0.2% SLS at 120 min (Y_2_) was given in Figure 1 and Figure 2, respectively by the three-dimension response surface.

In case of Y_1_, response surface plot showed that when X_1_ is lower than 0.6, Y_1_ does not increase with increasing X_2_ and X_3_ values. These results can be interpreted as PVP in the milling composition has more dominant effect on solubility and intrinsic dissolution rate of CXB. Additionally, if the X_3_ is lower than 1.0, it is necessary to have higher amount values of X_1_ to increase Y_1_.

In case of Y_2_, when X_2_ is higher than 0.10, higher values of X_1_ increase Y_2_. This shows that, unlike Y_1_, CXB is weakly acidic and therefore requires higher rates of surfactant to increase its intrinsic dissolution rate in acidic medium. However, higher rates of X_2_ are not enough to increase Y_2_, X_1_ and X_2_ should be at optimum rates for higher Y_2_. The ratio required for X_3_ to prevent CXB from hydrogen bonding with PVP should not suppress the ratio for PVP to increase solubility of CXB.

#### Model Validation and Optimum Composition

**Figure 3:**
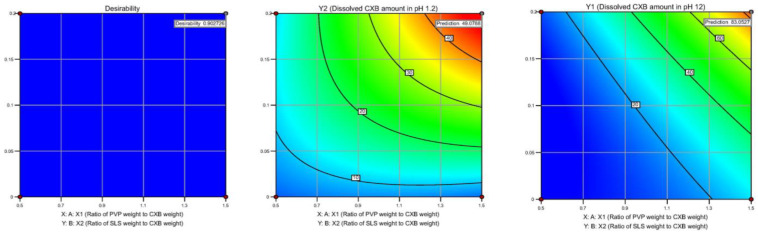
Contour plot of model validation.

As a result of factorial design experiment, the most influential formulation variables, and interaction of them were investigated at three levels. In our study, the desired CQA were set as >70 mg/cm^2^ intrinsic dissolution rate in pH 12 at 30 min and >40 mg/cm^2^ intrinsic dissolution rate in pH 1.2 + 0.2% SLS at 120 min. After the design space was created, the optimum formulation with a desirability function was determined. Thanks to the knowledge and experience gained via the design space, ratio of PVP/CXB was set at the highest level of 1.5, ratio of SLS/CXB was set between 0.15 to 0.20, and the ratio of MAN/CXB was set at three level of lowest, zero and highest. Experimental and predicted results of these configurations with error and desirability values were given in Table 6 and Figure 3. The dissolved amount in FASSIF medium was evaluated in addition to pH 12 and pH 1.2 + 0.2% SLS to determine optimum composition. This optimal condition was determined as follows: the ratio of PVP/CXB, SLS/CXB and MAN/CXB were 1.5, 0.2, 0.5, respectively.

### 3.2. Bayesian Optimization (BO)

BO offers an iterative and data-driven approach for selecting the experiments and optimizing a response variable of interest as described in detail in Section 2.4.2. [31]. The use of BO with real time experiments for optimization of CXB formulation provides an innovative approach for the drug formulation domain. The results regarding the cumulative experiment design matrix proposed by BO and the response variable Y_2_ are given in Table 7. According to the BO results, the acquisition function did not change much in the X_1_ and X_2_ variables, and it followed the “exploitation” strategy in the X_3_ variable, starting from BO07. BO scanned the range of 1.56–2 for X_1_, the range 0.14–0.175 for X_2_, and the range 0.5–1.95 for X_3_, starting from BO07 with the strategies of “exploit” and “explore” to reach the optimum formulation. From BO07 onwards, it was observed that BO suggested formulation compositions that are very close to the optimum co-milled CXB composition, which was also determined by CCD.

BO acquisition functions explores the results of different factor values in the first six experiments, and then they start to exploit the highest function points by narrowing down the changes in the suggested experiments. The results of CCD and the contour graphs are shown in Section 3.1. The formulations designed after the first six formulations with BO have designs very similar to the optimum formulations suggested by the CCD. In the CCD, the optimum formulation can be determined after the statistical evaluation of the data of 20 experiments; BO started to recommend formulations close to the optimum formulation design from the 15th experiment (eight experiments used from CCD to initialize BO and the cumulative evaluation of the following seven experiments with BO). These findings indicate that BO have the potential to optimize the response variable with less experimentation than the commonly used quadratic experimental designs.

### 3.3. Physicochemical Characterization of Co-Milled Celecoxib Composition

#### 3.3.1. Apparent Solubility

**Figure 4:**
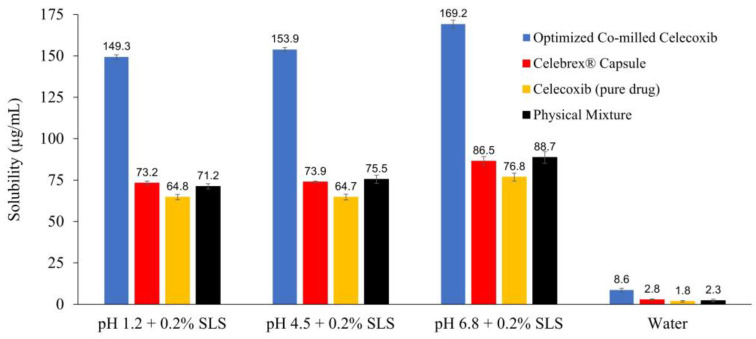
Apparent solubility of CXB pure powder, PM, Celebrex^®^ capsule, and optimized co-milled CXB in different media (n = 3, mean ± SD).

Figure 4 shows apparent solubility of CXB pure powder, PM, Celebrex^®^ capsule, and optimized co-milled CXB in pH 1.2, pH 4.5, pH 6.8 with 0.2%SLS, pH 12 and water. The apparent solubility of optimized co-milled CXB was significantly increased, approximately 2-fold (149.3 ± 1.36 µg/mL vs. 64.8 ± 1.61 µg/mL) in pH 1.2 + 0.2% SLS; 2-fold (153.9 ± 1.13 µg/mL vs. 64.7 ± 1.79 µg/mL) in pH 4.5 + 0.2% SLS; 2-fold (169.2 ± 2.33 µg/mL vs. 76.8 ± 2.37 µg/mL) in pH 6.8 + 0.2% SLS; over 4.8-fold (8.6 ± 1.06 µg/mL vs. 1.8 ± 0.33 µg/mL) in water when compared with CXB pure powder. Additionally, optimized co-milled CXB solubility in all media including pH 1.2, pH 4.5, pH 6.8, pH 12 and water was higher than commercial product of Celebrex^®^ capsule. On the other hand, the solubility of PM had acceptable values due to effect of excipient existing in milling composition. The solubility of it in water was 2.3 ± 0.62 µg/mL, while CXB pure powder was 1.8 ± 0.33 µg/mL.

#### 3.3.2. Intrinsic Dissolution Rate (IDR)

**Figure 5:**
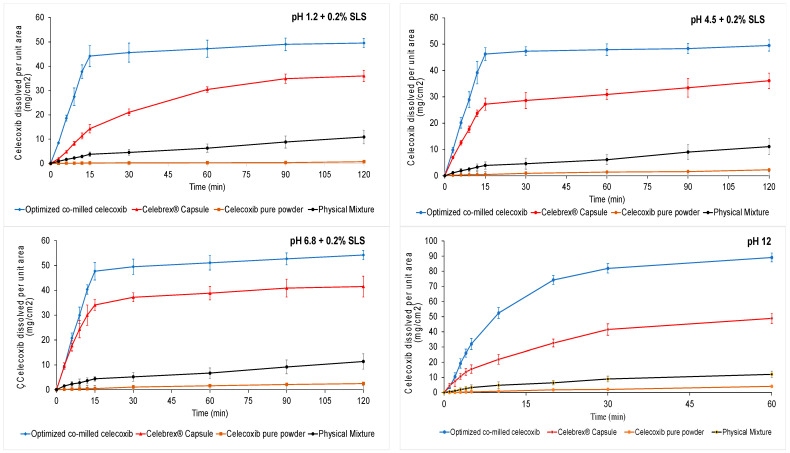

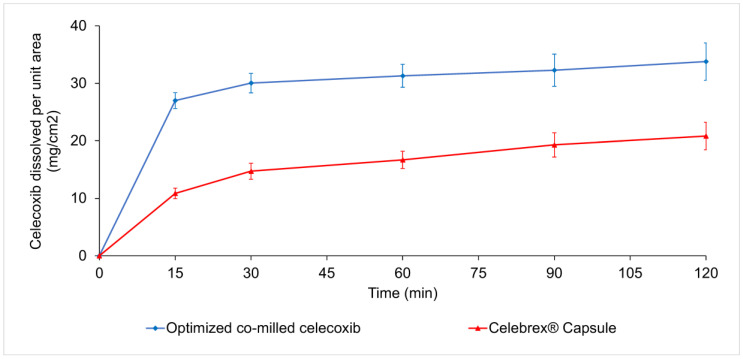
Intrinsic dissolution rate of CXB pure powder, PM, Celebrex^®^ capsule, and optimized co-milled CXB in different media.

Figure 5 shows IDR graphics of CXB pure powder, PM, Celebrex^®^ capsule, and optimized co-milled CXB in pH 1.2, pH 4.5, pH 6.8 containing 0.2% SLS, pH 12. The sampling time points were selected as the first five minutes in pH 12 due to fast release. In addition to these media, FASSIF medium was used to compare dissolution profiles (Figure S9: IDR regression graph and equation of regression).

The IDR of optimized co-milled CXB was significantly increased, approximately 203-fold (6.13 ± 0.5 mg/min/cm^2^ vs. 0.0296 ± 0.003 mg/min/cm^2^) in pH 1.2 + 0.2% SLS (Figure 5); 123-fold (6.03 ± 0.6 mg/min/cm^2^ vs. 0.0487 ± 0.002 mg/min/cm^2^) in pH 4.5 + 0.2% SLS (Figure 5); and 96-fold (6.43 ± 0.4 mg/min/cm^2^ vs. 0.0667 ± 0.002 mg/min/cm^2^) in pH 6.8 + 0.2% SLS (Figure 5); when compared with celecoxib pure powder.

The IDR of CXB was increased by the effect of dry co-milling process and excipients in the milling composition. The IDR results of PM and CXB pure powder were found to be similar. The difference of results was acceptable due to effect of excipient existing in milling composition. The intrinsic dissolution rate of Celebrex^®^ capsule was significantly higher than CXB pure powder. The reason of the high results could be the high amount of SLS in the Celebrex^®^ capsule formulation, the wet granulation method enhancing wettability of granules, and the super disintegrant providing the fast disintegration of granules as a result the quick interaction of medium and powder surface. However, the optimized co-milled CXB dissolution rate was higher than Celebrex^®^ capsule in all media. The IDR of optimized co-milled CXB was approximately 2.9-fold (6.13 ± 0.5 mg/min/cm^2^ vs. 2.09 ± 0.3 mg/min/cm^2^) in pH 1.2 (Figure 5); 1.8-fold (6.03 ± 0.6 mg/min/cm^2^ vs. 3.45 ± 0.5 mg/min/cm^2^) in pH 4.5 (Figure 5); and 1.6-fold (4.09 ± 0.4 mg/min/cm^2^ vs. 0.0667 ± 0.002 mg/min/cm^2^) in pH 6.8 (Figure 5) when compared with Celebrex^®^ capsule.

The dissolution rate in pH 12 was so fast when compared to the other dissolution media due to the high solubility in pH 12 (Figure 5). The IDR of optimized co-milled CXB was approximately 2.6-fold (14.45 ± 1.0 mg/min/cm^2^ vs. 5.61 ± 1.0 mg/min/cm^2^ vs), 10-fold (14.45 ± 1.0 mg/min/cm^2^ vs. 1.38 ± 0.4 mg/min/cm^2^) and 71-fold (14.45 ± 1.0 mg/min/cm^2^ vs. 0.20 ± 0.04 mg/min/cm^2^) when compared with Celebrex^®^ capsule, PM, and CXB pure powder, respectively.

Figure 5 shows the IDR profiles of CXB pure powder, PM, Celebrex^®^ capsule, and optimized co-milled CXB in FASSIF medium over a time course of 120 min. After 15 min, the dissolved amount in cm^2^ was 54 mg for optimized co-milled CXB while it was 22 mg for Celebrex^®^ capsule. Additionally, the maximum dissolved amount of optimized co-milled CXB was 61 mg at 120 min while it was 41 mg for Celebrex^®^ capsule. As a result, this dissolution rate difference in a biorelevant medium, which was simulated fasted intestinal medium, exhibited similar differences in in vivo media and maintain much higher amounts of CXB in systemic circulation.

#### 3.3.3. Differential Scanning Calorimetry (DSC)

**Figure 6:**
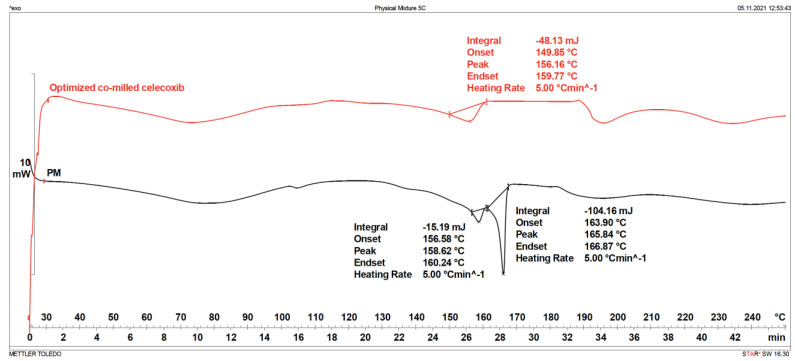
DSC thermograms of optimized co-milled CXB and PM.

It is a clear possibility that transforming from crystalline state to amorphous state by dry milling can happen [60,61]. Co-milling with PVP is the method of preparing amorphous solid dispersion, and it is expected that there exist no melting peaks in amorphous state. In preliminary screening experiments, it was determined that amorphous state was formed in the co-milling with PVP. However, it was hypothesized that the possibility of hydrogen bonding of CXB and PVP to form an amorphous solid dispersion was reduced because of the addition of mannitol to the milling composition. The DSC thermograms of PM, SLS, optimum co-milled CXB composition and PVP were given Figure S10. Onset, peak, and endset values were compared in DSC analyses of the PM and optimum composition at three different temperatures elevation rates: 5 °C/min, 10 °C/min and 15 °C/min. The fact that melting endotherms at different temperature increase rates (onset) are close to each other indicates that the investigated endotherm is the melting endotherm. In the thermograms of PM, the melting endotherms of CXB and MAN appear separately, although they are not exactly compatible with the pure states of these two substances (this is expected since they are mixtures). Onset, peak, and endset values of the melting endotherm observed at 5 °C/min, 10 °C/min and 15 °C/min in the thermogram for the optimum composition were close to each other (Figure S11: DSC thermograms of optimized co-milled CXB and PM at different temperature increase rate). Values related to this melting endotherm are thought to be related to CXB since it is close to the melting temperature of CXB. This explains the reason why MAN, and CXB, were not observed separately in thermograms for optimum composition as the molecular interaction between CXB, PVP, and MAN, and the fact that CXB was partially dissolved in this mixture. The obtained thermograms shows that CXB does not completely transform into amorphous form [39,62]. XRD (Figure S12: X-ray diffraction patterns of optimum co-milled CXB, PM, and MAN) and FTIR (Figure S13: FTIR spectrums of each excipient, PM and optimum co-milled CXB; Figure S14: FTIR spectrums of optimized co-milled CXB and PM) analyses also support this situation. Differential scanning calorimetry studies were performed for the evaluation of physical state of optimized co-milled CXB and PM, as shown in Figure 6.

#### 3.3.4. Powder X-ray Diffraction Analysis (PXRD)

**Figure 7:**
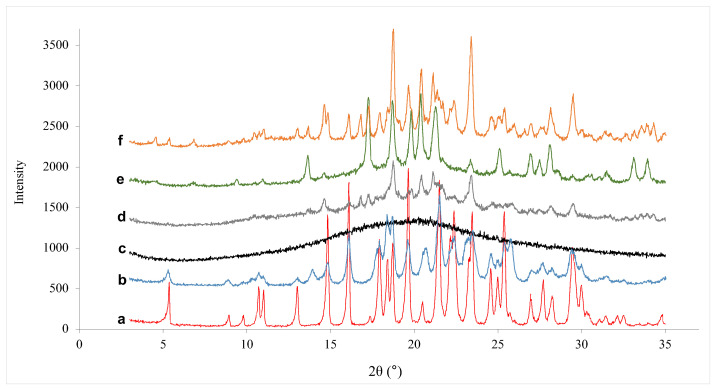
X-ray diffraction patterns of: (a) CXB pure powder, (b) milled CXB, (c) co-milled CXB with PVP, (d) optimum co-milled CXB, (e) co-milled CXB with PVP and MAN (MAN > PVP), and (f) PM.

CXB pure powder composed of crystal form-III was used in all experiments. Characteristic peaks of crystal CXB were at 16.0, 19.6, 21.5, 22.3, 23.4, 25.3, and 29.4°, while characteristic peaks of the crystal form-III were at 5.3, 10.7, 11.0, 13.0, 14.8, 16.1, 17.9, 18.4, 18.7, 19.6, 21.5, 22.1, 22.4, 23.4, 25.3, and 29.5°. Several solid dispersion manufacturing methods, such as antisolvent precipitation, spray drying, and milling, tend to create partial and/or full amorphization and crystalline transformation [19]. According to the literature, after manufacturing solid dispersion with PVP and crystalline CXB, crystalline form of CXB transforms to amorphous form. The crystal form-III milled CXB, and co-milled CXB with PVP are shown in Figure 7a–c, respectively. After co-milling of CXB and PVP, crystalline form-III transformed to completely amorphous form. However, when the mannitol was added to the co-milling composition, crystalline to amorphous transformation was prevented. Figure 7d belongs to co-milling composition of MAN < PVP and, the characteristic peak of crystal CXB was maintained. However, the intensity of peaks was decreased due to reduced particle size below 500 nm. Figure 7e demonstrates the co-milling composition of MAN > PVP and, the characteristic peak of crystal CXB was maintained, too. According to Bhatt et al., mannitol aids in nanocrystal generation by heterogeneous nucleation and providing physical barrier to crystal growth [63]. Therefore, it was necessary to use mannitol in our study not only to enhance solubility, but also to prevent amorphous conversion. In the screening experiments, XRD of co-milled compositions having the highest solubility was investigated. PVP had a significant effect on increasing of solubility, but when it was used individually with CXB, the amorphous CXB was transformed. The amorphous form carries the risks for stability problems in shelf life despite it has higher solubility and bioavailability than crystalline form. Because of these reasons, it was aimed to provide crystalline-amorph transformation at minimum level with selected co-milling composition.

#### 3.3.5. Particle Size and Scanning Electron Microscopy (SEM)

**Figure 8:**
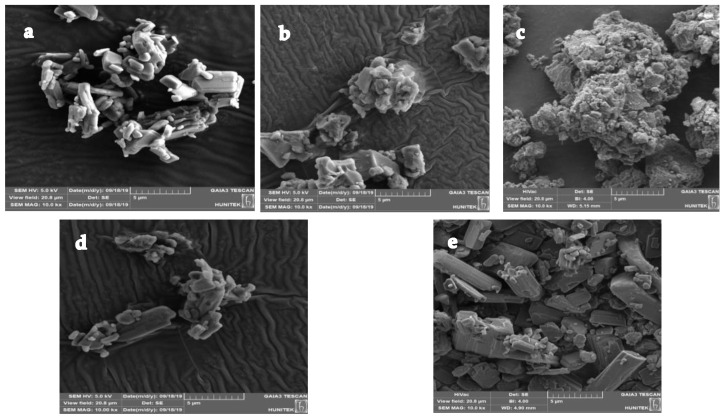
SEM micrographs of: (**a**) celecoxib pure powder, (**b**) co-milled celecoxib with PVP and SLS, (**c**) optimum co-milled celecoxib, (**d**) co-milled celecoxib with PVP, SLS and MAN (MAN > PVP), and (**e**) PM.

SEM micrographs of CXB pure powder and co-milled CXB are shown in Figure 8. CXB pure powder was in crystalline form having an average particle size distribution within the range of 1.4–7.0 µm (Figure 8a). The addition of PVP and SLS to the co-milling composition resulted in a significant reduction in average particle size and the two excipients acted as stabilizers, preventing the particles from aggregating. As a result, although the crystal structure of the particles decreased, the particles had more homogeneous morphology (Figure 8b). As a result of the addition of mannitol to the milling composition in addition to PVP and SLS, the change in the crystalline form of the particles decreased (Figure 8c). However, if the ratio of mannitol in the milling composition was higher than PVP, the change in the crystalline form is expected to be reduced more (Figure 8d). In the PM of the co-milling composition, the CXB particles were larger in size and in crystalline form (Figure 8e).

#### 3.3.6. Storage Stability

The results of stability studies are given in Table 8. The particle size and PDI were increased at the end of three months, but they are still lower than 300 nm and 0.5, respectively. The particle size and PDI of PM were found to be stable at the end of three months at both stability conditions. An assay of CXB in co-milled and PM was stable and there was no significant decrease. It can be concluded that excipient composition and manufacturing method of optimized formulation was accurately justified for a stable formulation. Unlike an assay of CXB, IDR results were decreased, when compared to those at initial time. The decrease in dissolved amount at 40 °C, 75% RH was higher than 25 °C, 60% RH for optimized co-milled mixture and PM. This is thought to be related to the high humidity condition. All results suggested that optimized co-milled CXB had a good stability, and it can provide high solubility and dissolution rate during shelf-life storage.

Polymorphic transformation and stability were also evaluated with XRD studies. XRD pattern in Figure S15 shows that there was no significant difference at characteristic peaks of CXB. The optimized co-milled CXB and PM were found to be stable at the end of three months at both stability conditions (Figure S15: X-ray diffraction patterns of optimized co-milled celecoxib: (a) initial time, (b) third month at 40 °C, 75% RH, (c) third month at 25 °C, 60% RH, and physical mixture, (d) initial time, (e) third month at 40 °C, 75% RH, (f) third month at 25 °C, 60% RH.

### 3.4. In Vitro Dissolution Study

**Figure 9:**
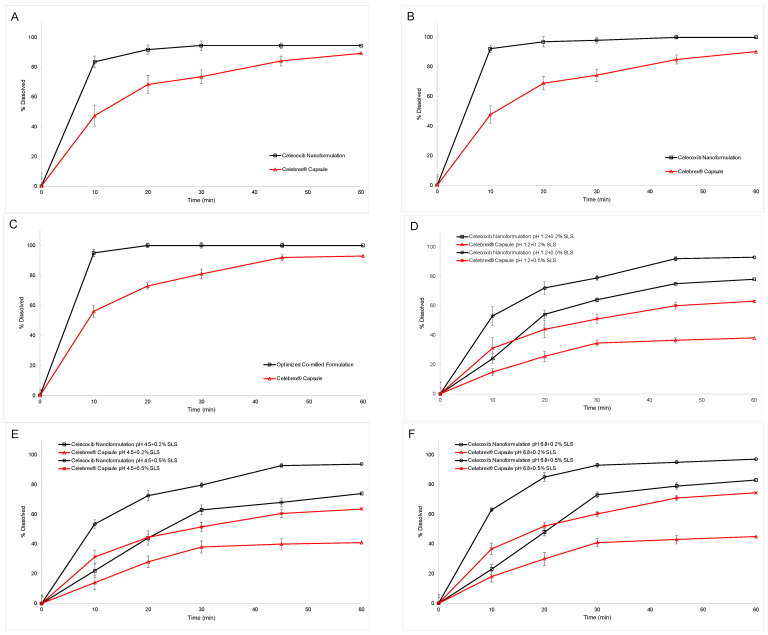
In vitro dissolution profiles of celecoxib nanoformulation and Celebrex^®^ capsule in different dissolution media (sink condition: (**A**): pH 1.2 + 0.2%, (**B**): pH 4.5 + 0.2%, (**C**): pH 6.8 + 0.2%; non-sink condition: (**D**): pH 1.2 + 0.2% and 0.5%, (**E**): pH 4.5 + 0.2% and 0.5%, (**F**): pH 6.8 + 0.2% and 0.5%).

The in vitro dissolution profiles of CXB nanoformulation and Celebrex^®^ capsule in four different media including pH 1.2, pH 4.5, pH 6.8, and pH 12, are given in Figure 9. The in vitro dissolution rate of formulations was investigated in both sink and non-sink conditions. CXB had poor solubility in water and physiological media. Therefore, dissolution studies of CXB formulation as a quality control analysis were conducted in pH 12 containing 1% SLS. However, the existence of SLS in dissolution medium not only insufficiently simulated the in vivo dissolution fluid, but also overshadowed the difference of formulations. Thus, dissolution studies were performed with different SLS concentrations. Firstly, according to the apparent solubility studies, the optimum CXB strength was determined for dissolution condition, which was 1000 mL pH 1.2, pH 4.5, pH 6.8 containing 0.2% SLS. As a result, 37 mg of CXB for nanoformulation and 19 mg of CXB for Celebrex^®^ capsule were used. Figure 9A–C displays the comparative dissolution profile in sink condition. Dissolution rate in pH 1.2, pH 4.5, and pH 6.8 were similar, when sink condition was maintained. The celecoxib nanoformulation had faster dissolution rate of 84 ± 5.1%, 92 ± 5.5%, 95 ± 3.5% in 10 min when compared with Celebrex^®^ capsule 47 ± 9%, 48 ± 7.2%, 56 ± 4.3% in pH 1.2, pH 4.5 and pH 6.8, respectively. Both samples dissolved more than 90% in 60 min in all three media.

In a second study, the capsule containing 200 mg CXB was tested in 1000 mL pH 1.2, pH 4.5, pH 6.8 containing 0.2% SLS and 0.5% SLS, and 1000 mL pH 12 containing 0.5% SLS. Figure 9D–F displays the comparative dissolution profile in non-sink condition The percentages of dissolution for CXB nanoformulation were 78 ± 0.8%, 74 ± 2.9%, 83 ± 2.1% in 60 min in 0.2% SLS pH 1.2, pH 4.5 and pH 6.8, respectively. On the other hand, percentages of dissolution of Celebrex^®^ capsule were 38 ± 1.9%, 41 ± 3.7%, 45 ± 2.9% in 60 min in 0.2% SLS pH 1.2, pH 4.5 and pH 6.8, respectively. The difference of these two samples showed that the CXB nanoformulation had high solubility and dissolution rate. When the same dissolution study was made with 0.5% SDS, the cumulative dissolved amount was increased for both samples. However, the difference of two samples were maintained, and test drug had faster dissolution rate in all media when compared with Celebrex^®^ capsule.

### 3.5. Cell Culture

The cytotoxicity evaluations of CXB pure powder, PM, Celebrex^®^ capsule, and CXB nanoformulation were determined using Caco-2 cells by incubating them with different concentrations of the samples for 4 h. The percentages of cell survival for samples at 1 to 1000 µM concentration are given in Figure S16: the percentage of cell survival for celecoxib pure powder, physical mixture, Celebrex^®^ capsule, and celecoxib nanoformulation at different concentrations of the samples for 4 h (mean ± SD, n = 6). After the determination of samples’ cytotoxicity and the selection of the optimum concentration to provide suitable cell viability, the permeability studies were conducted. In this study, the results of TEER measurements (>400 ohmxcm^2^) showed that the cell monolayers were intact, and no cellular damage occurred. The permeability result of pure CXB powder in drug suspension was 0.52 ± 0.15 × 10^−6^ cm/s while permeability values were 12.40 ± 3.12 × 10^−6^ cm/s, 9.36 ± 1.61 × 10^−6^ cm/s, 3.32 ± 0.69 × 10^−6^ cm/s for CXB nanoformulation, Celebrex^®^ capsule, and PM, respectively (Table S8: Mean P_app_ of Celecoxib in the Direction of Apical to Basolateral (mean ± standard deviation, n = 3). The permeability value of the CXB was found to be much lower than the permeability reported in the literature, which may be a consequence of administered concentration, dosage form, and the Caco-2 cells. Obtaining the highest permeability value with the nanoformulation is associated with the nanoformulation having the highest dissolved CXB amount among the suspension samples [43,64,65,66,67]. As a general expectation, the solubility of drug molecules is explained by the ionized form, while their passage through cell membranes is explained by the non-ionized form. In this case, since CXB has high permeability, the amount of dissolved CXB in the prepared suspension samples had a determining effect on the amount of substance that will pass through the cell membrane during the transition from the apical surface to the basolateral. It is thought that the higher permeability value of Celebrex^®^ capsule than the PM and active substance may be due to the increased wettability of the granules because of wet granulation with SLS, as in the intrinsic dissolution rate and dissolution tests.

### 3.6. In Vivo Oral Bioavailability

**Figure 10:**
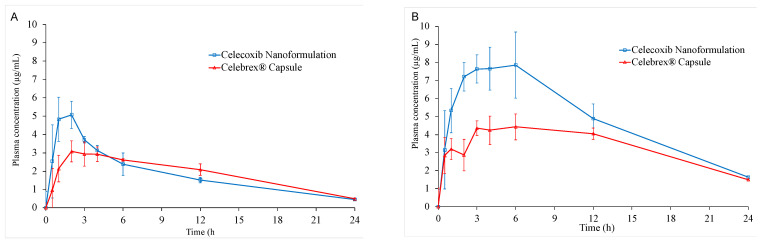
Plasma concentration vs. time profiles of CXB after a single oral administration of CXB nanoformulation and Celebrex^®^ capsule at a dose of 10 mg/kg (**A**) and 40 mg/kg (**B**). Data are presented as mean ± SD (n = 5).

The oral bioavailability values of CXB in CXB nanoformulation and Celebrex^®^ capsule were evaluated in rats. Two different doses which were 10 mg/kg and 40 mg/kg of CXB were administrated via gastric gavage. The comparative plasma concentration-time profile of CXB after oral administration of CXB nanoformulation and Celebrex^®^ capsule are presented in Figure 10 and the associated pharmacokinetic parameters are summarized in Table 9. The plasma concentration profile for CXB nanoformulation represented significant improvement in drug absorption when compared to the Celebrex^®^ capsule. However, the enhancement of bioavailability for 40 mg/kg (Figure 10A) oral dose was higher than 10 mg/kg dose (Figure 10B).

For 10 mg/kg administration dose, the C_max_ values of CXB nanoformulation and Celebrex^®^ capsule were 5.51 ± 1.06 μg/mL and 3.42 ± 0.35 μg/mL, respectively. Moreover, the C_max_ of CXB nanoformulation increased 1.61-fold compared to that of Celebrex^®^. The AUC_0–24_ values of celecoxib nanoformulation and Celebrex^®^ capsule were 41.75 ± 5.27 μg/mL and 41.97 ± 4.27 μg/mL, respectively. The AUC_0–24_ of samples was very similar, although C_max_ of CXB nanoformulation was higher than Celebrex^®^. Therefore, the relative bioavailability of CXB nanoformulation was 99.5%, compared to Celebrex^®^.

For 40 mg/kg administration dose, the C_max_ values of CXB nanoformulation and Celebrex^®^ capsule were 8.23 ± 0.82 μg/mL and 5.08 ± 0.38 μg/mL, respectively. Moreover, the C_max_ of CXB nanoformulation increased 1.62-fold compared to that of Celebrex^®^. The AUC_0–24_ values of CXB nanoformulation and Celebrex^®^ capsule were 110.94 ± 25.22 μg/mL and 76.42 ± 9.14 μg/mL, respectively. The relative bioavailability of CXB nanoformulation was 145.2%, compared to that of Celebrex^®^. In addition, CXB nanoformulation showed faster t_max_ 3.80 ± 2.28 h vs. 6.00 ± 3.67 h, indicating a more rapid absorption rate and higher absorption amount. In fact, in vitro dissolution of celecoxib was increased by co-milled composition, also resulting in increased oral bioavailability. This also implied that the oral bioavailability of CXB can be controlled by the in vitro dissolution and solubility properties. As the absorption of CXB took place throughout the gastrointestinal tract, more rapid dissolution, and higher permeability of CXB may have caused its faster absorption.

## 4. Conclusions

The enhancement of solubility and dissolution properties of CXB by dry co-milling technology, which is an industrially applicable method, was chosen in this research study. The most suitable excipients for CXB in dry co-milling were determined by preliminary screening experiments. The lowest particle size and the highest intrinsic dissolution rate were obtained in milling with PVP, MAN, and SLS. It was observed that the dissolution rate of CXB increased more with the addition of mannitol to the milling composition, and there was less change in the polymorphic structure of CXB. CCD was used to optimize formulation parameters. The effects of the factors were evaluated with three-dimensional response surface graphics and contour graphics, and the optimum formulation composition providing the highest dissolution rate was determined. A total of 12 experiments were conducted with the different experimental designs that BO experimental design presented with the information learned from eight experiments on the CCD and created in each iteration, and the formulation components that provided the highest intrinsic dissolution rate were determined. As an alternative to the CCD, it was seen that the target can be achieved with fewer experiments. Optimized co-milled CXB compositions were characterized, and CXB nanoformulations were developed and experimental studies were performed for in vitro/in vivo evaluation. As a result of in vitro and in vivo studies, dry co-milling technology can be commercially viable approach to produce CXB products with enhanced solubility, dissolution rate, and oral bioavailability.

## 5. Patents

Pharmaceutical compositions prepared by dry milling method and containing celecoxib with increased dissolution rate, TR 2020/17034B, PCT/TR2021/050619, WO/2021/230849.

## Figures and Tables

**Table 1:** The factors and their levels used in CCD.

Independent Factors		Design Level	
Coded	Actual Parameters	Coded Value	Actual Value
X_1_	PVP/CXB weight ratio	−1.633	0.1835
		−1	0.5
		0	1
		+1	1.5
		+1.633	1.8165
X_2_	SLS/CXB weight ratio	−1.633	−0.0633
		−1	0
		0	0.1
		+1	0.2
		+1.633	0.2633
X_3_	MAN/CXB weight ratio	−1.633	0.1835
		−1	0.5
		0	1
		+1	1.5
		+1.633	1.8165

**Table 2:** Experiments to start the (Gauss Process-GP) model.

Independent Factors			Response Variables
X_1_	X_2_	X_3_	Y_2_
1	0.1	1	20.00
1	0.1	1	27.50
1.5	0.2	0.5	42.70
0.5	0.2	0.5	5.00
1.5	0	0.5	1.70
1	0.1	1	30.00
0.5	0	1.5	1.20
1.5	0.2	1.5	42.30

**Table 3:** The cumulative experiment design matrix proposed by BO.

Run Order	Formulation Code	Independent Factors		
		X_1_	X_2_	X_3_
1	BO01	1.23	0.14	0.79
2	BO02	1.92	0.02	0.64
3	BO03	0.81	0.19	1.13
4	BO04	1.63	0.15	1.04
5	BO05	1.44	0.16	0.9
6	BO06	1.55	0.05	1.28
7	BO07	1.99	0.175	0.81
8	BO08	1.56	0.156	0.85
9	BO09	1.85	0.16	1.95
10	BO10	1.7	0.16	0.9
11	BO11	1.9	0.16	1.2
12	BO12	2.0	0.14	0.5

**Table 4:** Observed response for the 20 experimental runs in central composite design.

Run Order	Formulation Code	Independent Factors			Response Variables	
		X_1_	X_2_	X_3_	Y_1_	Y_2_
1	CC01	1	0.1	1	90	42.7
2	CC02	1	−0.0633	1	56.8	1.5
3	CC03	1	0.1	0.1835	13.9	3.7
4	CC04	1	0.1	1	26	20
5	CC05	0.1835	0.1	1	8.7	1
6	CC06	1	0.1	1.8165	29	1.7
7	CC07	1	0.2633	1	24	1.2
8	CC08	1.8165	0.1	1	28	27.5
9	CC09	1	0.1	1	30	30
10	CC10	1	0.1	1	37.8	32.8
11	CC11	1.5	0.2	0.5	87	42.3
12	CC12	0,5	0.2	0.5	9.8	5
13	CC13	1.5	0	0.5	65.4	46
14	CC14	1	0.1	1	26.3	1.8
15	CC15	0.5	0	1.5	45.1	16.7
16	CC16	1.5	0.2	1.5	31.6	23.1
17	CC17	1.5	0	1.5	6.8	10.6
18	CC18	0.5	0	0.5	11.8	22
19	CC19	1	0.1	1	30	19.8
20	CC20	0.5	0.2	1.5	69.5	43.3

X_1_: PVP/CXB weight ratio; X_2_: SLS/CXB weight ratio; X_3_: MAN/CXB weight ratio; Y_1_: Dissolved CXB amount in pH 12; Y_2_: Dissolved CXB amount in pH 1.2 + 0.2% SLS.

**Table 5:** Summary of the ANOVA for responses Y_1_ and Y_2_ in the quadratic model.

	Y_1_: Dissolved CXB Amount in pH 12 after 30 min				Y_2_: Dissolved CXB Amount in pH 1.2 + 0.2% SLS			
	Sum of Squares	Degree of Freedom	F-Value	*p* Value	Sum of Squares	Degree of Freedom	F-Value	*p* Value
Model	11,565.2	9	32.96	<0.0001	143.23	1	11.76	0.0006
X_1_	7151.9	1	183.42	<0.0001	4361.28	9	31.096	0.0003
X_2_	1599.8	1	41.03	0.0001	1281.16	1	46.88	<0.0001
X_3_	728.8	1	18.69	0.0019	1931.49	1	0.19	0.6691
X_1_X_2_	1255.0	1	32.19	0.0003	8.04	1	17.20	0.0025
X_1_X_3_	3.6	1	0.09	0.7667	708.76	1	0.00	0.9786
X_2_X_3_	220.5	1	5.66	0.0414	0.03	1	0.01	0.9274
X_1_^2^	135.4	1	3.47	0.0953	0.36	1	1.21	0.2986
X_2_^2^	489.7	1	12.56	0.0063	50.14	1	3.07	0.1139
X_3_^2^	2.5	1	0.06	0.8056	126.28	1	7.38	0.02376
Residual	350.9	9			304.09	1		
Lack of Fit	269.6	5	2.65	0.1829	370.79	9	2.29	0.2212
Pure Error	81.3	4			274.79	5		
Cor Total	11,920.4	19			96.01	4		
	R^2^ = 0.9706				R^2^ = 0.9216			

X_1_: PVP/CXB weight ratio; X_2_: SLS/CXB weight ratio; X_3_: MAN/CXB weight ratio.

**Table 6:** The experimental and the predicted results of the optimum co-milled CXB composition based on desirability function.

Formulation Trial				Predicted and Experimental Results		
	X_1_	X_2_	X_3_		Y_1_	Y_2_
1	1.6	0.15	1.0	Predicted	77.3	46.6
				Experimental	83.2	49.1
				Error	7.63	5.3
				Desirability	0.92	
2	1.5	0.2	1.5	Predicted	88.6	47.2
				Experimental	87.0	42.3
				Error	1.8	10.3
				Desirability	0.99	
3	1.5	0.2	0.5	Predicted	83.1	49.1
				Experimental	90.0	47.2
				Error	8.3	3.8
				Desirability	0.90	

**Table 7:** The cumulative experiment design matrix proposed by BO and Y_2_ response results.

Formulation Code	Independent Factors			Response Variable
	X_1_	X_2_	X_3_	Y_2_
BO01	1.23	0.14	0.79	45.0
BO02	1.92	0.02	0.64	1.7
BO03	0.81	0.19	1.13	16.4
BO04	1.63	0.15	1.04	49.1
BO05	1.44	0.16	0.9	47.8
BO06	1.55	0.05	1.28	9.6
BO07	1.99	0.175	0.81	46.2
BO08	1.56	0.156	0.85	46.1
BO09	1.85	0.16	1.95	36
BO10	1.7	0.16	0.9	48.5
BO11	1.9	0.16	1.2	48
BO12	2.0	0.14	0.5	48.4

**Table 8:** Stability results of optimized co-milled celecoxib and physical mixture at 40 °C, 75% RH and 25 °C, 60% RH.

Optimized Co-Milled CXB	Time (Month)	Assay (%)	Dissolved CXB (mg/cm^2^)	Z-Average (nm)	PDI
40 °C, 75% BN	0	100.5	92.2	183.50	0.24
	1	101	85.8		
	2	98.7	83.2		
	3	98.2	77.2	219.47	0.30
25 °C, 60% BN	3	99.8	84.0	217.33	0.33
**PM**	Time (month)	Assay (%)	Dissolved CXB (mg/cm^2^)	Z-Average (nm)	PDI
40 °C, 75% BN	0	103	22.6	1438.33	0.21
	1	101	22.0		
	2	99.5	17.2		
	3	99.8	13.8	1406.67	0.30
25 °C, 60% BN	3	102	24.8	1493.67	0.25

**Table 9:** Pharmacokinetic parameters of CXB nanoformulation and Celebrex^®^ capsule after oral administration in rats (n = 5, mean ± SD).

PK Parameters	10 mg/kg		40 mg/kg	
	Celecoxib Nanoformulation	Celebrex^®^ Capsule	Celecoxib Nanoformulation	Celebrex^®^ Capsule
C_maks_ (µg/mL)	5.51 ± 1.06	3.42 ± 0.35	8.23 ± 0.82	5.08 ± 0.38
t_maks_ (h)	1.60 ± 0.55	2.60 ± 1.34	3.80 ± 2.28	6.00 ± 3.67
AUC_0–24_ (h·µg/mL)	41.75 ± 5.27	41.97 ± 4.27	110.94 ± 25.22	76.42 ± 9.14
AUC_0–∞_ (h·µg/mL)	46.81 ± 4.16	45.30 ± 6.82	142.45 ± 41.83	99.27 ± 6.04
t_1/2_ (h)	7.35 ± 1.45	6.45 ± 1.46	9.91 ± 4.47	10.40 *
MRT (h)	7.93 ± 0.39	9.21 ± 0.98	9.33 ± 0.89	10.44 ± 0.42
k_a_ (h^−1^)	2.38 ± 1.28	1.76 ± 1.52	2.00 ± 1.60	0.96 *
k_e_ (h^−1^)	0.10 ± 0.02	0.11 ± 0.02	0.08 ± 0.03	0.07 *

* SD was not given as the value could be calculated from a single rat.

## Data Availability

Not applicable.

## Supplementary Materials

The following supporting information can be downloaded at: https://www.mdpi.com/article/10.3390/pharmaceutics15020363/s1, Table S1: Dissolved amount of celecoxib in pH 12 and average particle size results; Table S2: Dissolved amount of celecoxib in pH 1.2 + 0.2% SLS; Figure S1: XRD diffractograms for celecoxib, F01, F03, F20, and F21; Figure S2: FTIR spectra for celecoxib and F03; Figure S3: Ishikawa diagram of celecoxib co-milling process; Figure S4: Bayesian Optimization flow chart; Table S3: Plackett-Burman design and results; Table S4: Statistical analysis of dependent variable values obtained by Plackett-Burman experimental design; Figure S5: USP Wood Apparatus components, sample preparation; Table S5: Celecoxib Nanoformulation and Celebrex^®^ capsule qualitative and quantitative composition; Figure S6: Images of drug administration (A) and tail vein blood collection (B and C) in rats; Table S6: Chromatographic conditions for the celecoxib assay method; Table S7: MS/MS detection conditions for the celecoxib assay method; Figure S7: Contour plots for Y1, Figure S8: Contour plots for Y_2_; Figure S9: IDR regression graph and equation of regression; Figure S10: The DSC thermograms of physical mixture, SLS, optimum co-milled CXB composition and PVP; Figure S11: DSC thermograms of optimized co-milled celecoxib and physical mixture at different temperature increase rate; Figure S12: X-ray Diffraction patterns of optimum co-milled celecoxib, PM and mannitol; Figure S13: FTIR spectra: (a) CXB (b) MAN, (c) PVP, (d) SLS, (e) optimum co-milled celecoxib composition, and (f) physical mixture for optimum composition; Figure S14: FTIR spectrums of optimized co-milled celecoxib and physical mixture; Figure S15: X-ray diffraction patterns of optimized co-milled celecoxib: (a) initial time (b), third month at 40 °C, 75% RH, (c) third month at 25 °C, 60% RH, and physical mixture: (d) initial time (e), third month at 40 °C, 75% RH, (f) third month at 25 °C, 60% RH; Figure S16: Percentage of cell survival for celecoxib pure powder, physical mixture, Celebrex^®^ capsule, and celecoxib nanoformulation at different concentrations of the samples for 4 h (mean ± SD, n = 6); Table S8: Mean Papp of Celecoxib in the Direction of Apical to Basolateral (mean± standard deviation, n = 3).
