# Supplementary material for: Celecoxib Nanoformulations with Enhanced Solubility, Dissolution Rate, and Oral Bioavailability: Experimental Approaches over In Vitro/In Vivo Evaluation
Source: Pharmaceutics. 2023 Jan 20;15(2):363. doi: 10.3390/pharmaceutics15020363 (PMC9964073; doi:10.3390/pharmaceutics15020363)
Supplement: Supplementary file 1 [file pharmaceutics-15-00363-s001.zip › pharmaceutics-2128219-supplementary.pdf]

Table S1. Dissolved amount of celecoxib in pH 12 and average particle size results.

Formulation Code	Milling Composition	CXB/Excipient Weight Ratio	Dissolved amount of CXB in pH 12 at 60 min.	Particle Size (nm)
F01	CXB	1	2	606
F02	CXB:SLS	1:0,2	9	494
F03	CXB:PVP K12	1:1	100	486
F04	CXB:PVP K12:SLS	1:1:0,2	100	420
F05	CXB:PVP K30	1:1	90	502
F06	CXB:PVP K90	1:1	86	512
F07	CXB:LHPC LH 21	1:2	1	1020
F08	CXB:HEC	1:2	14	894
F09*	CXB:HEC:SLS	1:2:0,2	-	-
F10*	CXB:HEC:PS40	1:2:0,2	-	-
F11*	CXB:HEC:SLS:PS40	1:2:0,2:0,2	-	-
F12	CXB:LAC	1:2	5	596
F13	CXB:LAC:SLS	1:2:0,2	11	357
F14	CXB:LAC:SLS:PS40	1:8,6:0,2:0,2	20	425
F15	CXB:MAS	1:5	1	485
F16	CXB:PVP-VA	2:1	107	640
F17	CXB:PVP-VA:SLS	2:1	110	595
F18	CXB:SOL	1:1	13	487
F19	CXB:MAN	1:1	4	1120
F20	CXB:PVP:MAN	1:1	110	950
F21	CXB:PVP:MAN:SLS	1:1,5:0,5:0,2	120	350
F22	CXB:PVP:MAN:SLS	1:0,5:1,5:0,2	105	720

*There is a chemical reaction between celecoxib and excipient. The products could not be analysed.

CXB: celecoxib, SLS: sodium lauryl sulfate, PVP: polyvinylpyrrolidone, L-HPC LH-21: low-substituted hydroxypropyl cellulose, HEC: hydroxyethyl cellulose, PS40: polyoxyl 40 stearate, LAC: lactose monohydrate, MAS: magnesium alumino metasilicate, PVP-VA: vinylpyrrolidone and vinyl acetate copolymer, SOL: Soluplus®, MAN: Mannitol.

Table S2. Dissolved amount of celecoxib in pH 1.2 + 0.2% SLS at 120 min.

Formulation Code	Dissolved amount of celecoxib in pH 1.2 + 0.2% SLS at 120 min.
CXB pure powder	0.5
F03	2
F14	12.5
F20	15
F21	50
F22	30

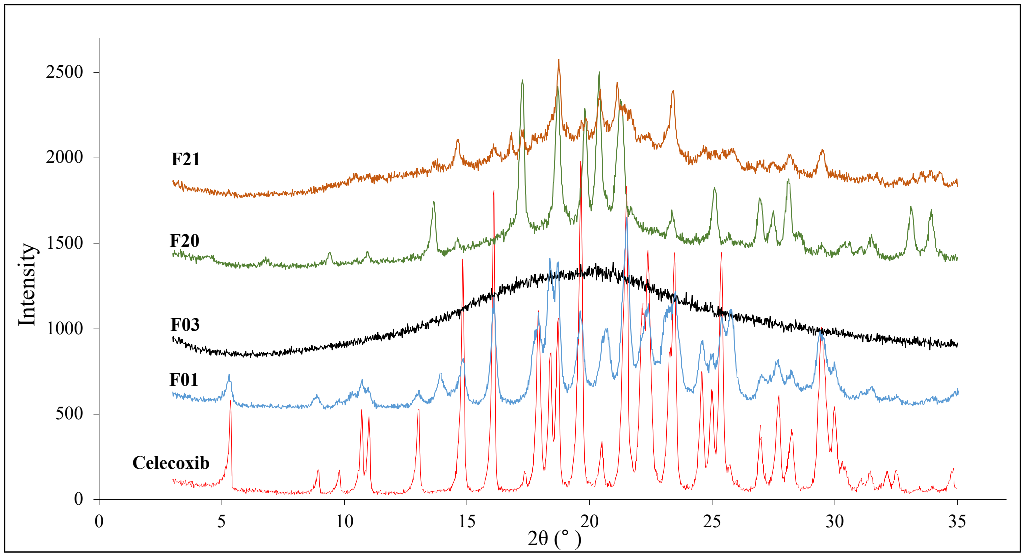

Figure S1. XRD diffractograms for celecoxib, F01, F03, F20, and F21.

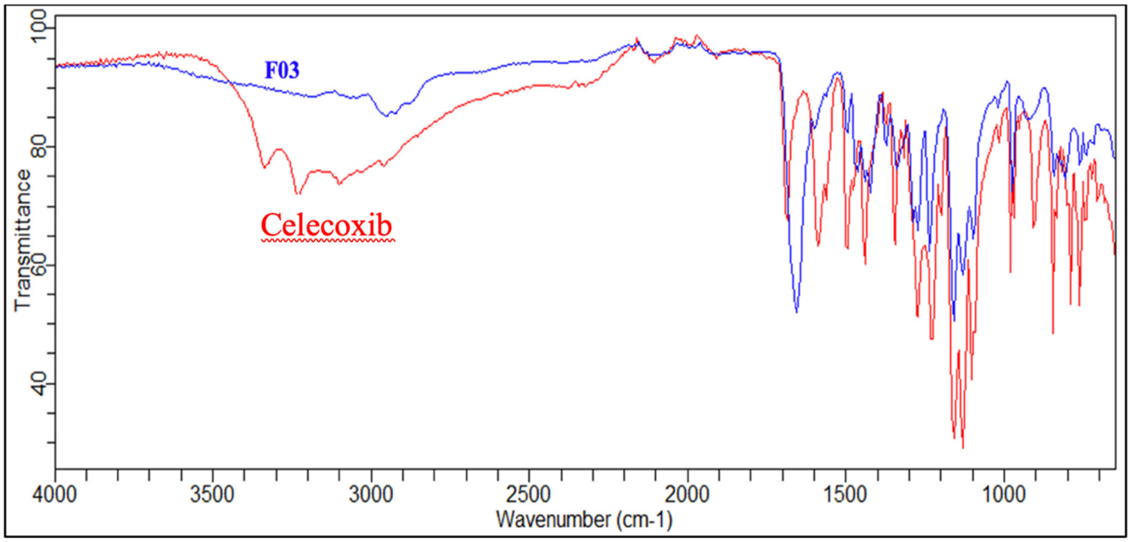

Figure S2. FTIR spectra for celecoxib and F03.

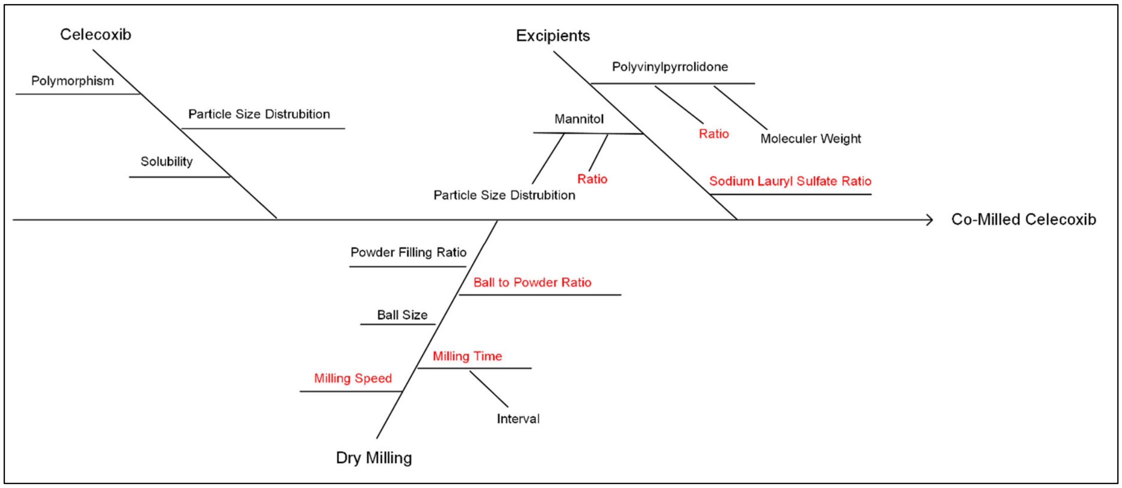

Figure S3. Ishikawa diagram of celecoxib co-milling process.

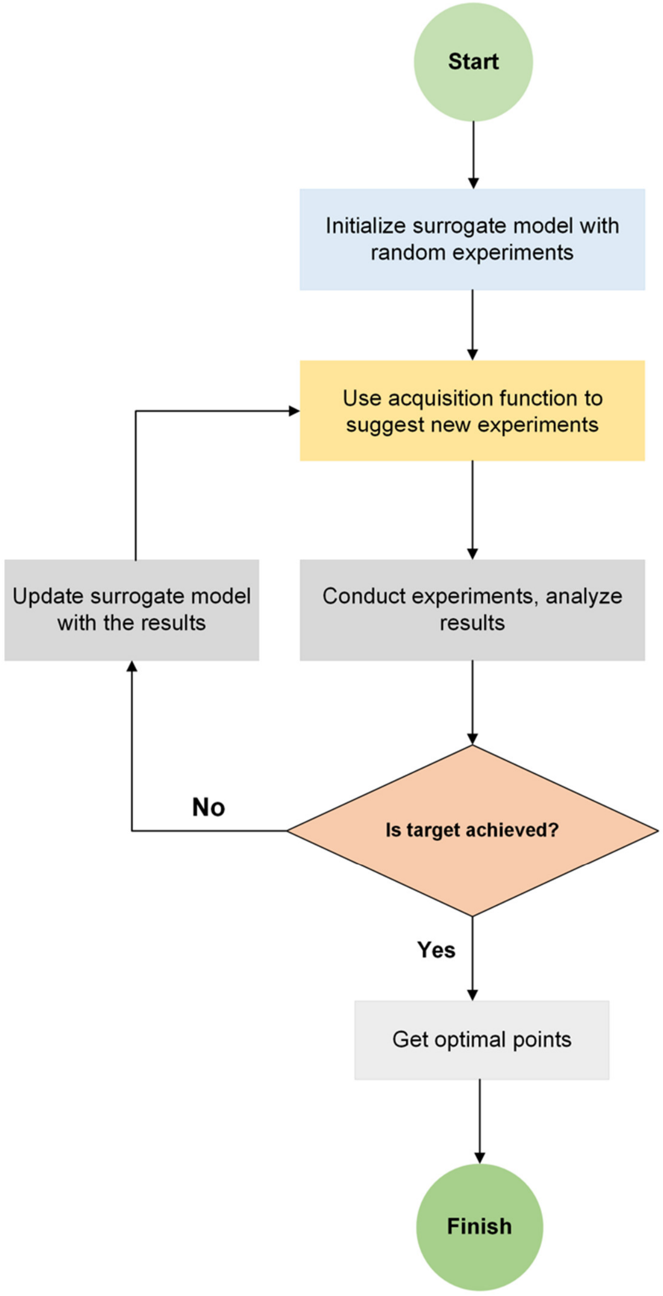

Figure S4. Bayesian Optimization flow chart.

Table S3. Plackett-Burman design and results.

Run Order	Formulation Code	X_1: Milling speed (rpm)	X_2: Milling time (h)	X_3: Ratio of ball weight to powder	X_4: Ratio of PVP/CXB	X_5: Ratio of SLS/CXB	X_6: Ratio of MAN/CXB	Y_1: Average particle size (nm)	Y_2: The dissolved amount in pH 12 at 30 min.	Y_3: The dissolved amount at pH 1.2 +0.2% SLS at 120 min.	Y_4: The apparent solubility in water (µg/mL)
1	PB01	250	1	5	0.5	0	0.5	980	4,1	0,8	2,4
2	PB02	500	4	5	1.5	0	0.5	650	28,1	1,3	2,6
3	PB03	500	1	15	1.5	0	1.5	821	67,5	5	3,1
4	PB04	250	4	5	0.5	0	1.5	1100	29,2	0,7	1,7
5	PB05	250	1	15	1.5	0.2	0.5	350	73	43,1	8,6
6	PB06	500	4	15	0.5	0.2	1.5	720	64,2	12	3,7
7	PB07	250	4	15	1.5	0	1.5	606	80,12	18,9	4,3
8	PB08	250	1	5	1.5	0.2	1.5	1240	36,42	42	6,9
9	PB09	500	1	15	0.5	0	0.5	645	18,11	1,2	2,5
10	PB10	250	4	15	0.5	0.2	0.5	487	17,3	5,9	3,1
11	PB11	500	4	5	1.5	0.2	0.5	677	23,21	5	3
12	PB12	500	1	5	0.5	0.2	1.5	1012	16,27	8	3,5

Table S4. Statistical analysis of dependent variable values obtained by Plackett-Burman experimental design.

	Y_1: Average particle size (nm)		Y_2: The dissolved amount in pH 12 at 30 min.		Y_3: The dissolved amount at pH 1.2 +0.2% SLS at 120 min.		Y_4: The apparent solubility in water (µg/mL)	
	Coefficient	*p* Value	Coefficient	*p* Value	Coefficient	*p* Value	Coefficient	*p* Value
Constant	705,9	0,039	38,13	0,042	11,99	0,046	3,783	0,041
X_1: Milling speed (rpm)	31,6	0,350	-1,90	0,660	-6,58	0,043	-0,717	0,070
X_2: Milling time (h)	17,4	0,594	2,23	0,607	-4,69	0,112	-0,717	0,070
X_3: Ratio of ball weight to powder	-101,1	0,022	15,24	0,013	2,36	0,377	0,433	0,223
X_4: Ratio of PVP:CXB	-68,1	0,077	13,26	0,022	7,23	0,031	0,967	0,027
X_5: Ratio of SLS:CXB	-11,1	0,732	0,27	0,949	7,34	0,030	1,017	0,022
X_6: Ratio of MAN:CXB	124,4	0,010	10,82	0,045	2,44	0,362	0,083	0,800

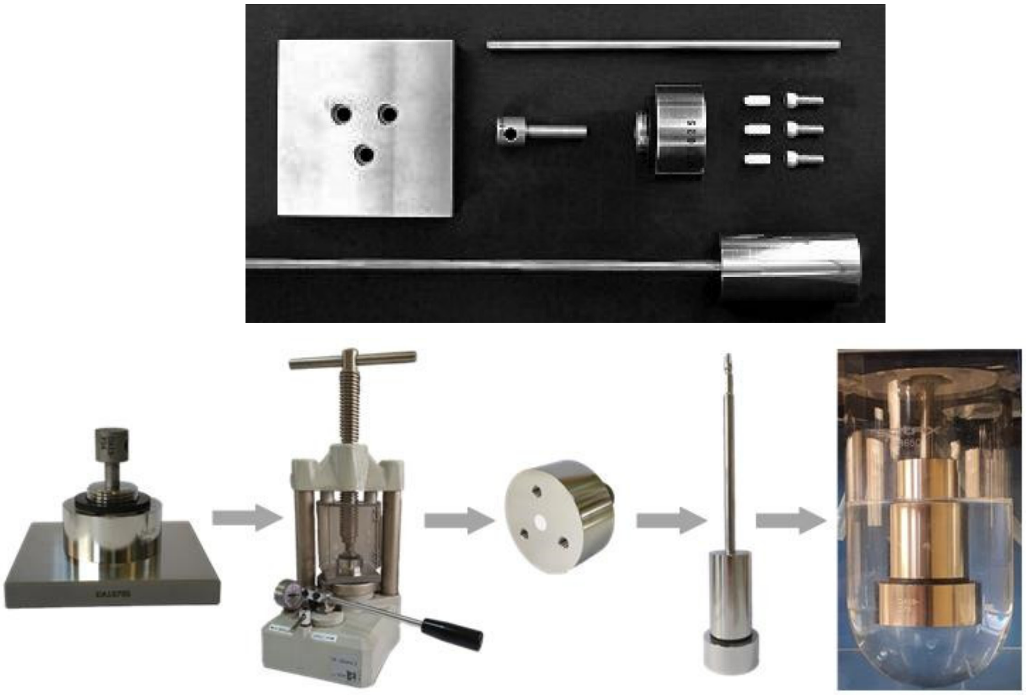

Figure S5. USP Wood Apparatus components, sample preparation.

Table S5. Celecoxib Nanoformulation and Celebrex® Capsule qualitative and quantitative composition.

Components	Celecoxib Nanoformulation (mg/capsule)	Celebrex® Capsule (mg/capsule)
Co-milled Celecoxib*	640	-
Celecoxib	-	200
Croscarmellose Sodium	8.1	*UNK*
Magnesium Stearate	2.7	*UNK*
Lactose monohydrate	-	49.8
Sodium Lauryl Sulfate	-	*UNK*
Polyvinylpyrrolidone	-	*UNK*

*It contains 200 mg of celecoxib

UNK: Unknown

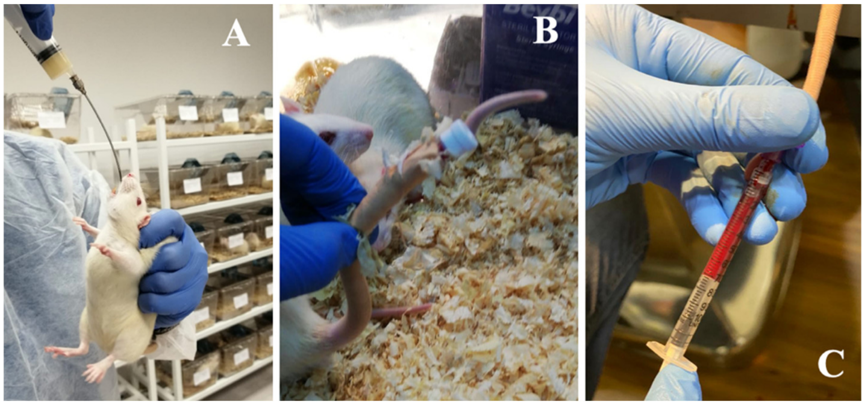

Figure S6. Images of drug administration (A) and tail vein blood collection (B and C) in rats

Table S6. Chromatographic conditions for the celecoxib assay method.

Column	:	C18; 350 mm x 2,1 mm, 3 µm
Flow rate	:	0.35 mL/min
Injection volume	:	10 µL
Column temperature	:	40°C
Sample temperature	:	Ambient
Run time	:	7 min
Mobile Phase	:	0.1% formic acid in water (mobile phase A) and 0.1% formic acid in acetonitrile (mobile phase B)
Gradient elüsyon		0-1 min: 70% mobile phase A<br>1-2 min: 70% mobile phase A<br>2-3 min: 30% mobile phase A<br>4-4.1 min: 30% mobile phase A<br>4.1-7 min: 70% mobile phase A

Table S7. MS/MS detection conditions for the celecoxib assay method.

Ionization	:	Negative
Interface voltage	:	4,5 kV
Nebulizer gas flow rate	:	3 mL/min
Drying gas flow rate	:	15 L/min
Desolvation line temperature	:	250°C
Heat block temperature	:	400°C
Multiple reaction monitoring (MRM)	:	*Celecoxib:* 380,0 $[M - H]^-$ → 316,0 *m/z*<br>Internal Standard: 557,20 $[M - H]^-$ → 397,1 *m/z*
Dwell time	:	*200 ms*
Collision energies (CE)	:	*Celecoxib*: 24 eV<br>Internal Standard: 31 eV

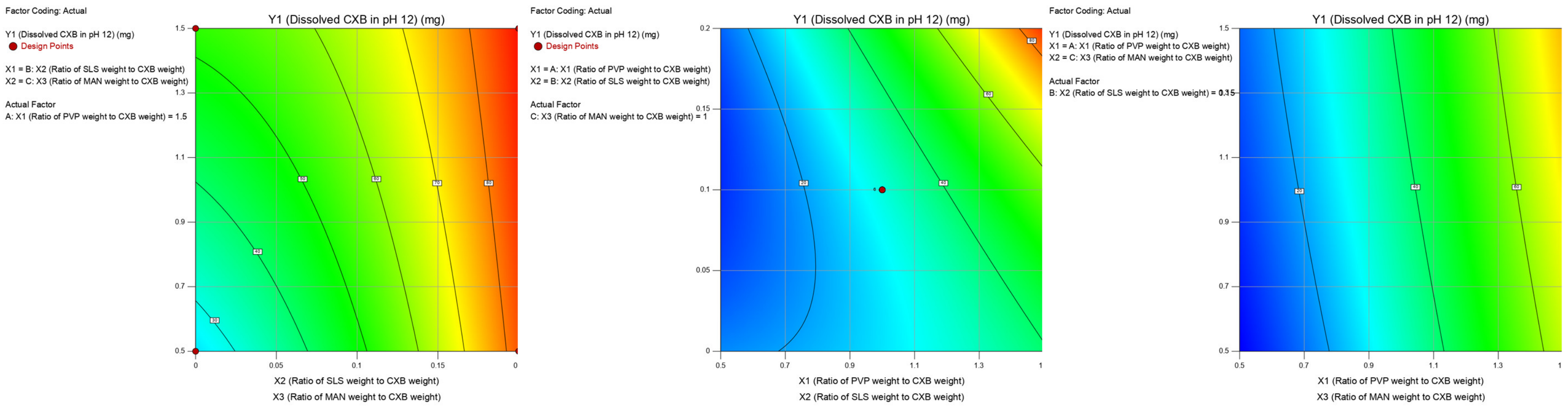

Figure S7. Contour plots for Y1.

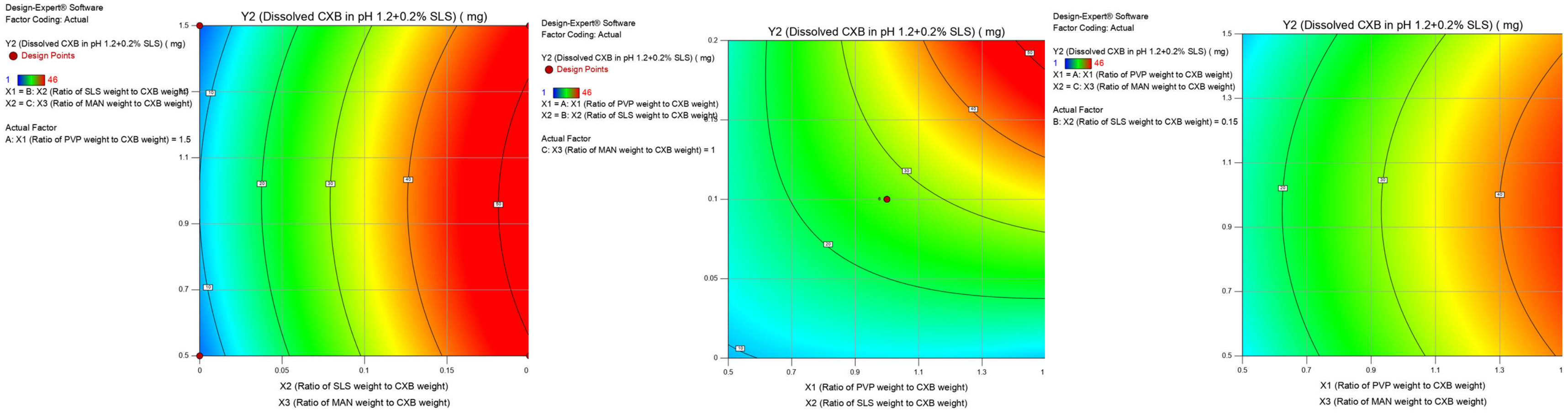

Figure S8. Contour plots for Y2.

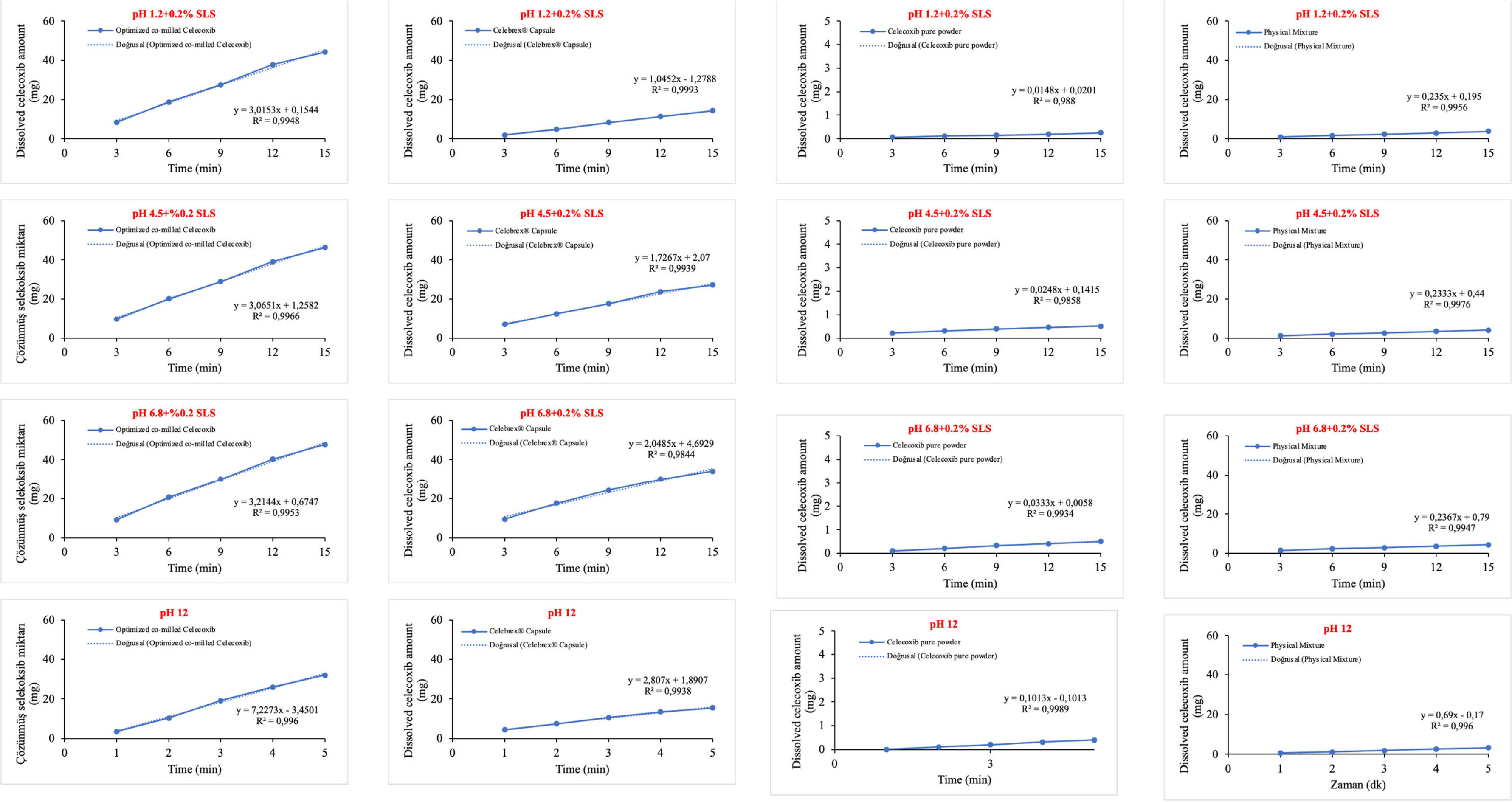

Figure S9. IDR regression graph and equation of regression.

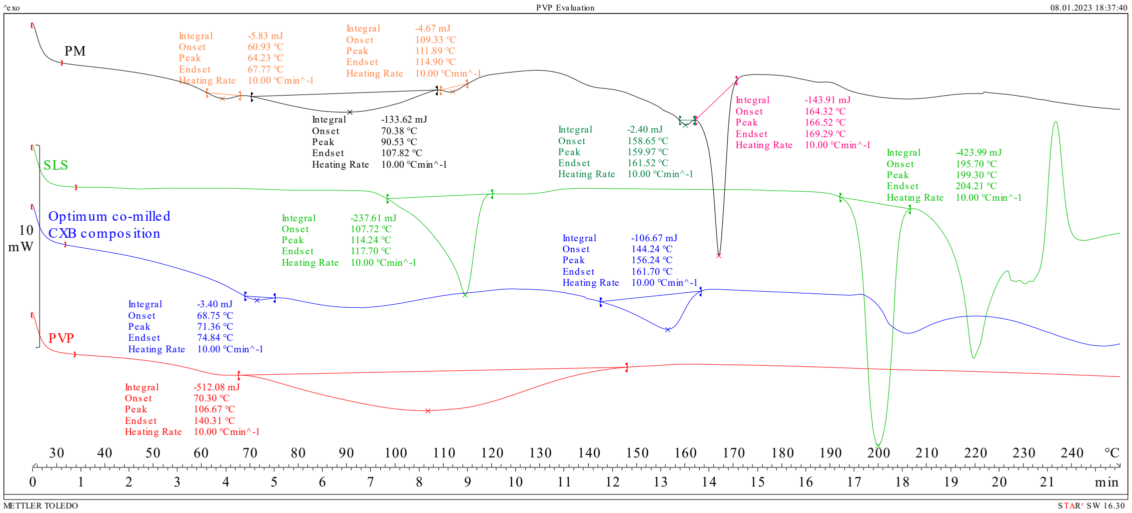

Figure S10. The DSC thermograms of physical mixture, SLS, optimum co-milled CXB composition and PVP.

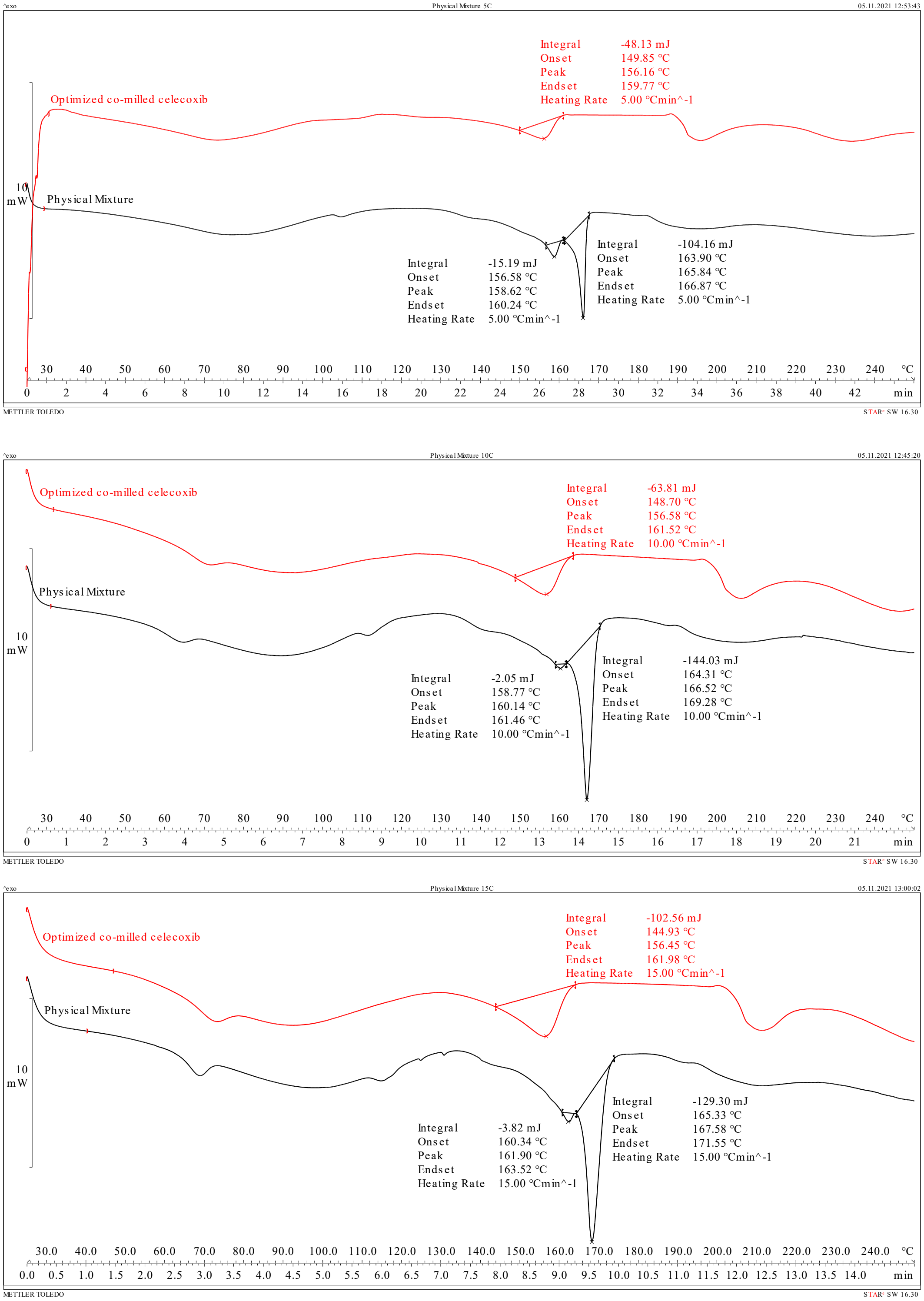

Figure S11. DSC thermograms of optimized co-milled celecoxib and physical mixture at different temperature increase rate).

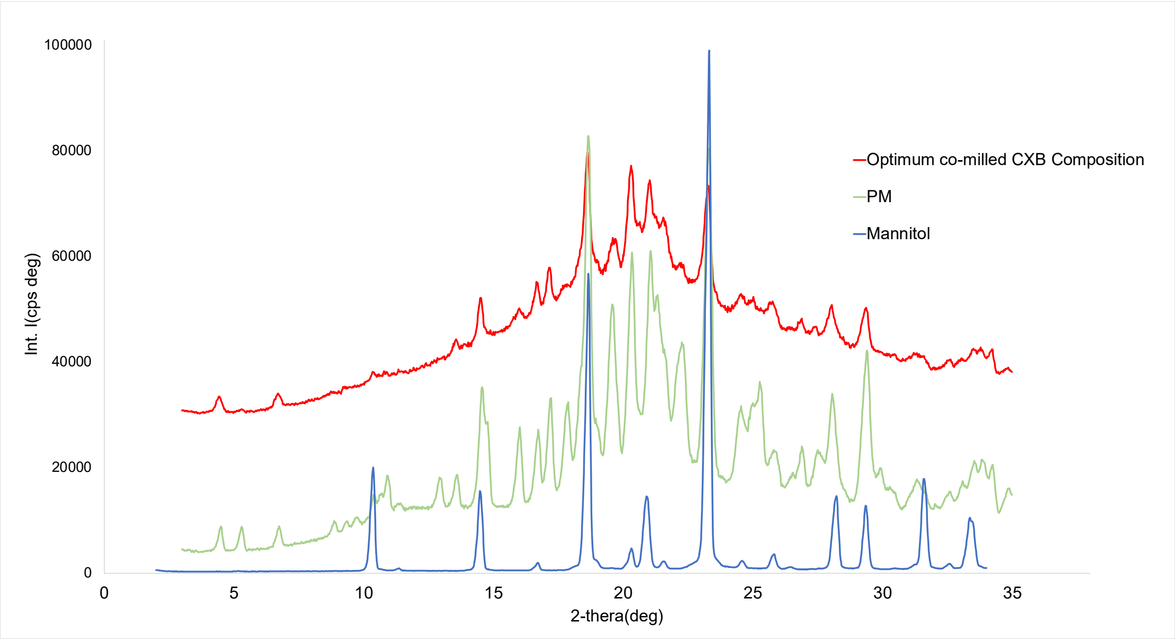

Figure S12. X-Ray Diffraction patterns of optimum co-milled celecoxib, PM and mannitol.

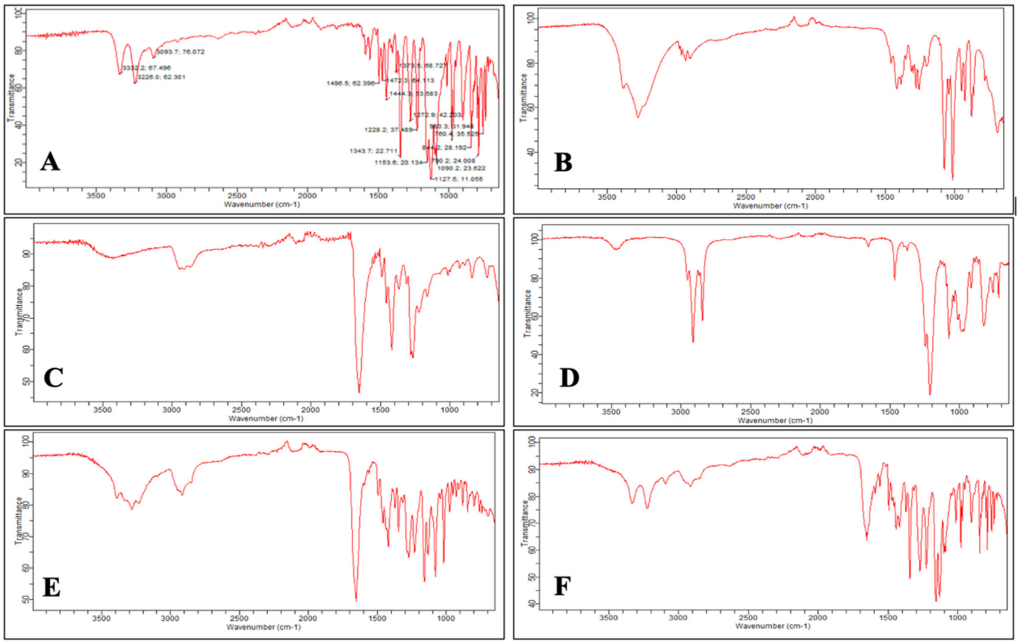

Figure S13. FTIR spectra: a) CXB b) MAN, c) PVP, d) SLS, e) optimum co-milled celecoxib composition, and f) physical mixture for optimum composition

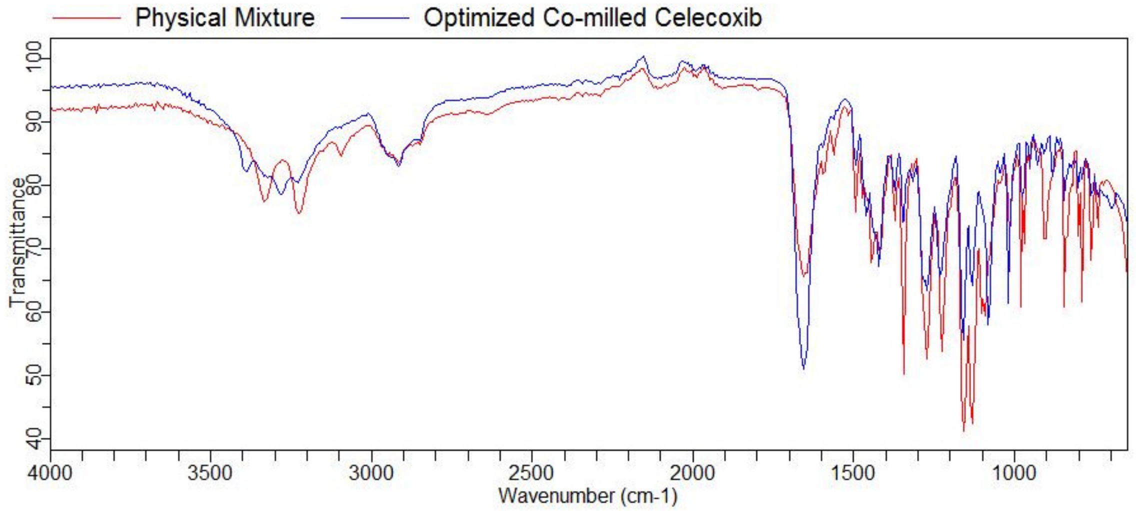

Figure S14. FTIR spectrums of optimized co-milled celecoxib and physical mixture.

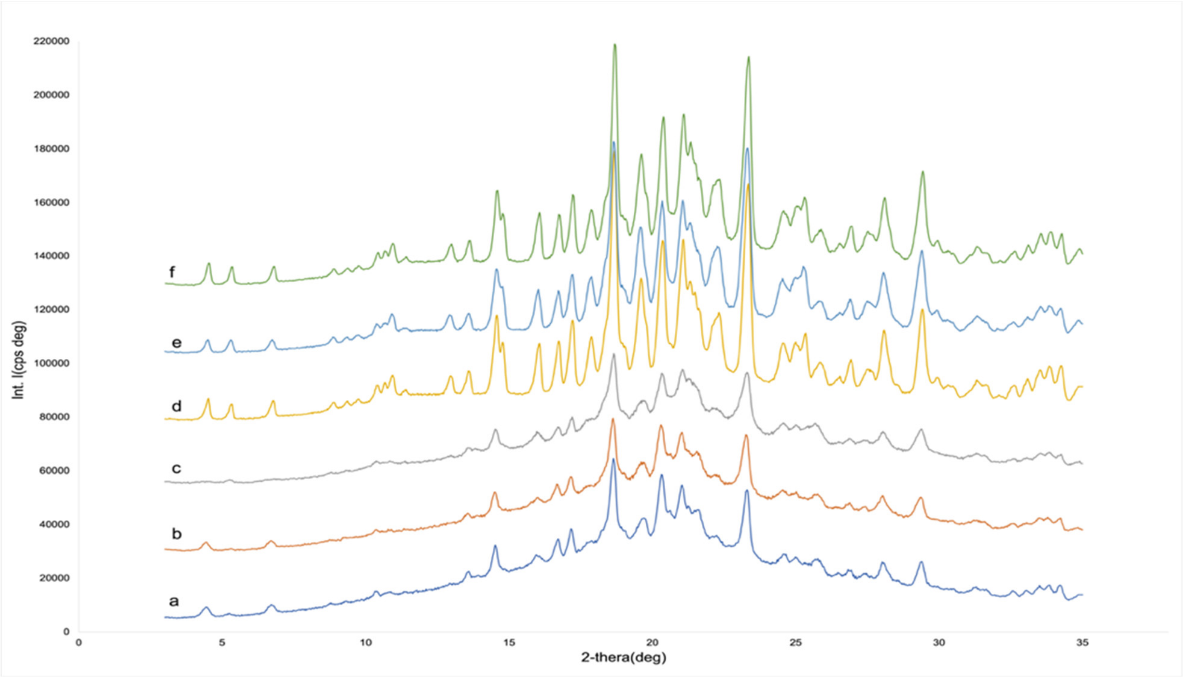

Figure S15. X-Ray Diffraction patterns of optimized co-milled celecoxib: (a) initial time (b), third month at 40°C, 75% RH, (c) third month at 25°C, 60% RH, and physical mixture: (d) initial time (e), third month at 40°C, 75% RH, (f) third month at 25°C, 60% RH).

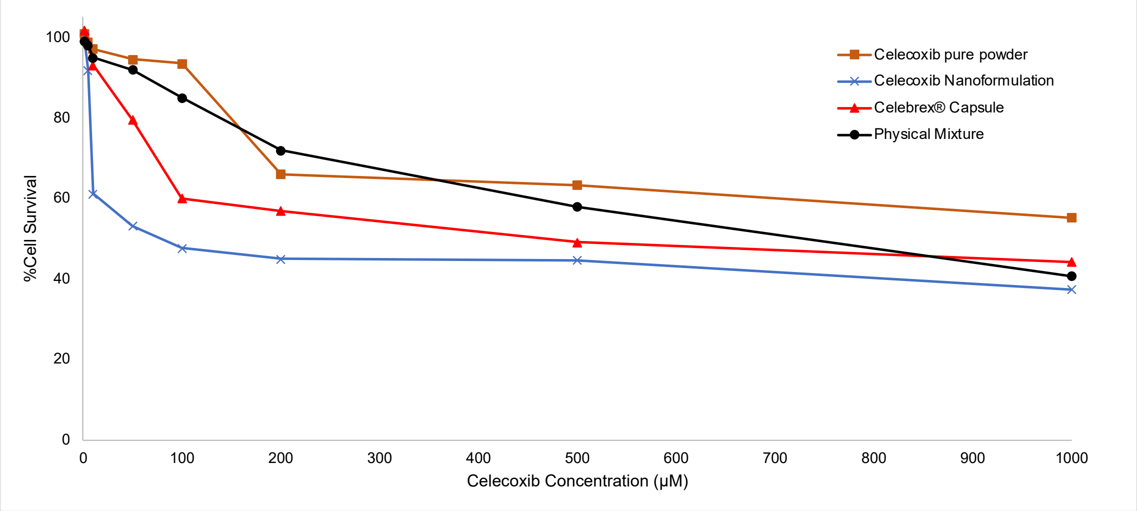

Figure S16. Percentage of cell survival for celecoxib pure powder, physical mixture, Celebrex® Capsule, and celecoxib nanoformulation at different concentrations of the samples for 4 h (mean ± SD, n=6).

Table S8. Mean Papp of Celecoxib in the Direction of Apical to Basolateral (mean± standard deviation, n=3).

**Formulation**	**Paap (cm/s x 10^{-6})<br>Absorptive (Apical to Basolateral)**
Celecoxib Nanoformulation	12,40±3.12
Celebrex® Capsule	9,36±1.61
Physical Mixture	3,32±0.69
Pure Celecoxib Powder	0,52±0,15
